# Multiple introductions and overwintering shape the progressive invasion of *Aedes albopictus* beyond the Alps

**DOI:** 10.1002/ece3.9138

**Published:** 2022-07-25

**Authors:** Laura Vavassori, Ann‐Christin Honnen, Norah Saarman, Adalgisa Caccone, Pie Müller

**Affiliations:** ^1^ Swiss Tropical and Public Health Institute Allschwil Switzerland; ^2^ University of Basel Basel Switzerland; ^3^ Department of Biology and Ecology Center Utah State University Logan USA; ^4^ Department of Ecology and Evolutionary Biology Yale University New Haven Connecticut USA; ^5^ Present address: Kantonales Laboratorium Basel‐Stadt Basel Switzerland

**Keywords:** Asian tiger mosquito, fine‐scale population genomics, human‐assisted dispersal, overwintering, recent invasion, skip oviposition

## Abstract

*Aedes albopictus* originates from Southeast Asia and is considered one of the most invasive species globally. This mosquito is a nuisance and a disease vector of significant public health relevance. In Europe, *Ae. albopictus* is firmly established and widespread south of the Alps, a mountain range that forms a formidable biogeographic barrier to many organisms. Recent reports of *Ae. albopictus* north of the Alps raise questions of (1) the origins of its recent invasion, and (2) if this mosquito has established overwintering populations north of the Alps. To answer these questions, we analyzed population genomic data from >4000 genome‐wide SNPs obtained through double‐digest restriction site‐associated DNA sequencing. We collected SNP data from specimens from six sites in Switzerland, north and south of the Alps, and analyzed them together with specimens from other 33 European sites, five from the Americas, and five from its Asian native range. At a global level, we detected four genetic clusters with specimens from Indonesia, Brazil, and Japan as the most differentiated, whereas specimens from Europe, Hong Kong, and USA largely overlapped. Across the Alps, we detected a weak genetic structure and high levels of genetic admixture, supporting a scenario of rapid and human‐aided dispersal along transportation routes. While the genetic pattern suggests frequent re‐introductions into Switzerland from Italian sources, the recovery of a pair of full siblings in two consecutive years in Strasbourg, France, suggests the presence of an overwintering population north of the Alps. The suggestion of overwintering populations of *Ae. albopictus* north of the Alps and the expansion patterns identified points to an increased risk of further northward expansion and the need for increased surveillance of mosquito populations in Northern Europe.

## Graphical abstract

Population genomics study to investigate the recent invasion of *Aedes albopictus* in Europe across the Alps. While we detected a weak genetic structure and high levels of genetic admixture, the recovery of a pair of full sibling consecutive years in Strasbourg, France, suggests the presence of an overwintering population north of the Alps. The expansion patterns points to an increased risk of further spread and the need for increased surveillance of mosquito population in Europe.

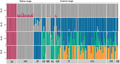

## 1 INTRODUCTION

Reconstructing the history of biological invasions is fundamental to understand the evolutionary and ecological processes underlying successful invasions (Estoup & Guillemaud, 2010). The genetic structure of invasive populations reflects their introduction history, which includes their geographic origin, the number of introduction events (i.e., propagule pressure), and the number of individuals initiating the invasion (Garnas et al., 2016; Lockwood et al., 2005). A lack of genetic variation is expected in invading populations as the founder populations are often constituted by a limited number of individuals and experience pronounced genetic drift (Dlugosch & Parker, 2008). If the genetic variation of the founder population is too low, it may not be able to establish in a new environment and thus, it will disappear eventually (Facon et al., 2006). Indeed, previous findings suggest that successful biological invasions often originate from multiple rather than single introduction events (Dlugosch & Parker, 2008; Lockwood et al., 2005). Multiple introductions contribute to maintaining high genetic diversity and population size of the invading populations (Cristescu, 2015). This is especially true if introductions originate from geographically distant sources, as it increases the probability of introducing individuals with different genetic backgrounds (Rius & Darling, 2014). Once established, connectivity among introduced populations can additionally lead to admixture that further increases genetic variation, and this, in turn, may increase the probability of successful establishment and, ultimately, further spread (Slatkin, 1985).

For invasive vector species, knowledge of their dispersal dynamics, source populations, and introduction pathways is not only of academic interest but also of immediate relevance for public health. Understanding the invasion history allows better estimates of the risk of establishment of new populations, and thus provides important information for monitoring and control (Estoup & Guillemaud, 2010). A great example of a successful biological invader is *Aedes albopictus* (Skuse, 1894), the Asian tiger mosquito. It is considered one of the most invasive species worldwide (Global Invasive Species Database, 2020). Due to its vector competence for several arboviruses, including chikungunya, dengue, and Zika (Gratz, 2004; Wong et al., 2013), as well as dirofilarial worms (Cancrini et al., 2003), *Ae. albopictus* is of particular public health concern.

*Aedes albopictus* eggs can resist desiccation for long periods and overcome lower temperatures during winter in temperate zones through diapause (Hanson & Craig, 1994). These biological factors greatly facilitated the global expansion of this mosquito species together with human activities, which contributed to its expansion by enabling dispersal over long and shorter distances. Like other invasive *Aedes* species, *Ae. albopictus* is passively spread across continents primarily through the international trade of used tires into which mosquitoes had deposited eggs before shipment (Paupy et al., 2009). At the regional level, adult mosquitoes frequently hitch ride in vehicles and are subsequently displaced along roads (Egizi et al., 2016; Medlock et al., 2015).

Over the last four decades, *Ae. albopictus* has spread to every continent except Antarctica, while its native distribution range is in Southeast Asia, from tropical (e.g., Indian Ocean Islands and Indonesia; Bonizzoni et al., 2013) to temperate regions (Japan; Kobayashi et al., 2002). In mainland Europe, *Ae. albopictus* was first recorded in Albania in 1979 (Adhami & Reiter, 1998). In the Americas, it was first reported from Texas, USA, in 1985 (Sprenger & Wuithiranyagool, 1986) and 1 year later from the State of Rio de Janeiro, Brazil (Oswaldo, 1986). The populations in North America are considered to have served as bridgehead populations for secondary introductions into Europe (Garnas et al., 2016; Lombaert et al., 2010) at two sites in Northern Italy between 1990 and 1991 (Dalla Pozza & Majori, 1992; Sabatini et al., 1990). From there, the mosquito quickly spreads across Southern Europe (Sherpa et al., 2019).

To date, *Ae. albopictus* has firmly established across the Mediterranean region from Spain to Greece (ECDC, 2019), and from the sea to the foot of the Alps (Flacio et al., 2016). In addition, modeling studies, considering present and future climatic conditions, suggest that its range will be expanding even further north (Caminade et al., 2012; Kraemer et al., 2019). Indeed, isolated populations of *Ae. albopictus* have already been reported from north of the Alps in Southern Germany (Becker et al., 2013; Pluskota et al., 2008; Werner et al., 2012) and northern Switzerland (Biebinger, 2020) with mosquitoes frequently re‐introduced across the Alps along the highways from south to north (Fuehrer et al., 2020; Müller et al., 2020). Given the very patchy pattern of the reported *Ae. albopictus* populations and the uncertainties of the climatic models, the extent to which local populations north of the Alps are actually self‐sustainable, rather than temporarily established by re‐introduced individuals, is uncertain and their origins also remain largely unknown.

High‐resolution population genetic markers are fundamental to accurately resolve invasion histories of target species, especially for species with a recent invasion on a fine geographical scale like *Ae. albopictus* (Cristescu, 2015). Previous studies attributed difficulties to reconstruct invasion histories to low resolution of genetic markers, such as mitochondrial DNA or microsatellites (Goubert et al., 2016; Manni et al., 2017). Genomic analysis based on the screening of thousands of genome‐wide single nucleotide polymorphisms (SNPs) using double‐digest restriction site‐associated DNA sequencing (ddRAD‐seq) allows for high‐resolution studies, enabling detection of patterns and levels of genetic differentiation for *Ae. albopictus* at different spatial resolutions ranging from global (Kotsakiozi et al., 2017), to continental (Pichler et al., 2019; Sherpa et al., 2019), and to city scales (Schmidt et al., 2017) studies. Here, we aimed at a higher resolution by using ddRADseq to identify a panel of 4000 SNPs to investigate the introduction of *Ae. albopictus* into Switzerland, to reconstruct the invasion history across the Alps and to evaluate if current populations are self‐sustained. The spatial scale of this study is about 300 km along the south–north axis across the Alps. To facilitate detection of both long‐ and short‐range dispersal events, we screened for genomic variations in specimens from six sites in Switzerland north and south of the Alps, 33 sites in Europe, 5 sites from the Americas, and 5 sites from its Asian native range. To evaluate temporal stability of the *Ae. albopictus* populations north of the Alps, we screened for variation in three population samples collected over two consecutive years.

## 2 MATERIALS AND METHODS

### 2.1 Sampling strategy

**FIGURE 1:**
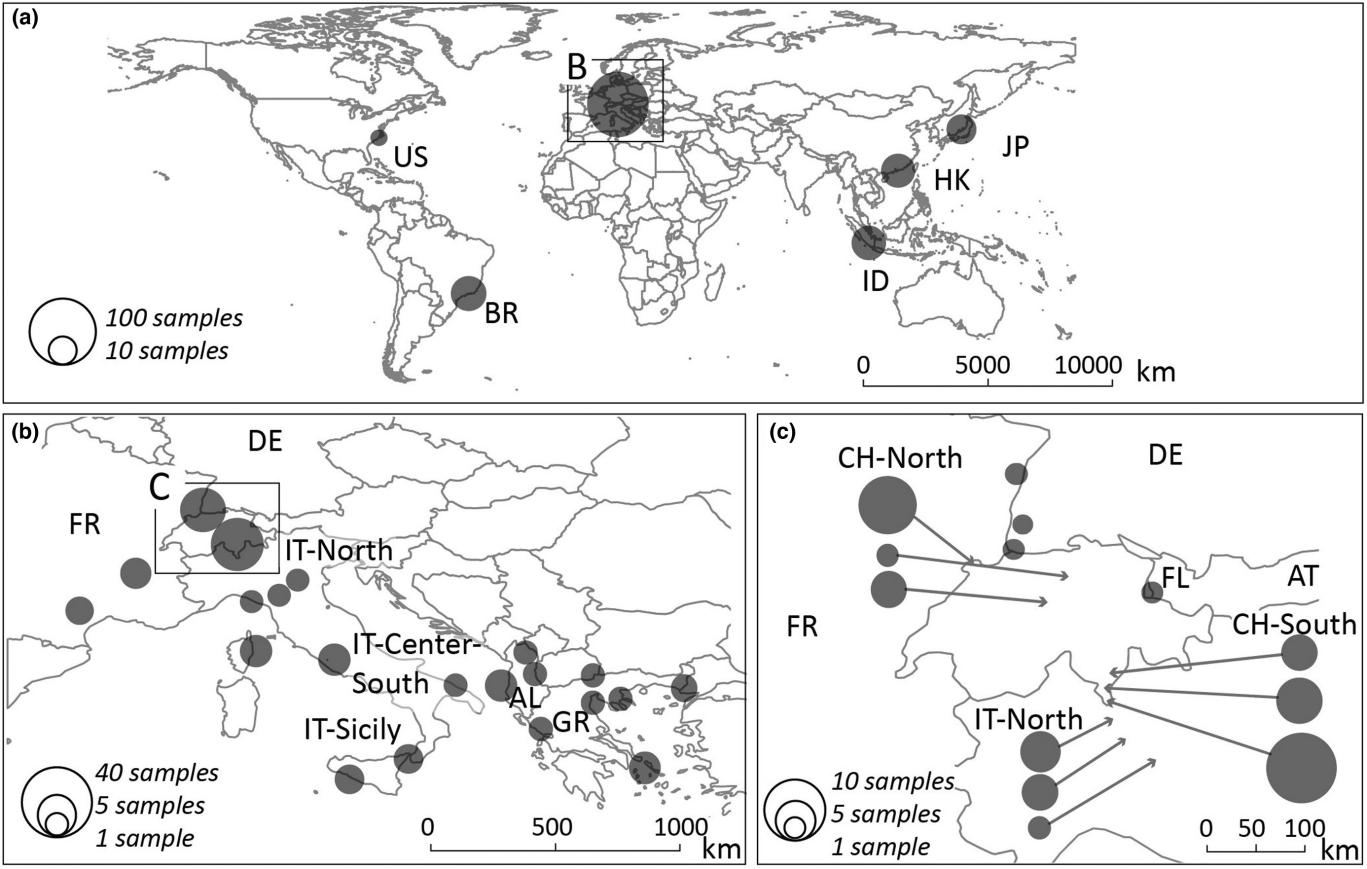
*Aedes albopictus* sampling sites. The pie charts represent collection sites, where the size of each pie represents how many individuals were collected in each location. The panels represent the sampling sites at the (a) global, (b) Europe, and (c) Swiss levels.

The sampling locations are reported in Table 1 and Figure 1, and all details on collection sites, time points, and methods are reported in Appendix S1 (Table S1). First, we investigated long‐range migration using a dataset consisting of 208 individuals from the native and invasive range (dataset named *1.native_invasive*). Second, we assess dispersal at the European scale and genetic structuring across the Alps using a dataset consisting of a subset of 137 individuals, which included only European samples from 39 sites (dataset named *2.europe*; Table 1 and Figure 1).

**TABLE 1:** *Aedes albopictus* specimens included in the two datasets of the present study

Dataset		Country	Collection sites (*N*)	*N*_ind_
*1.native_invasive*	*2.europe*	Switzerland (CH)	6	56
		Italy (IT)	15	34
		France (FR)	5	20
		Germany (DE)	3	9
		Liechtenstein (FL)	1	1
		Albania (AL)	4	8
		Greece (GR)	5	9
		Brazil (BR)	4	17
		USA (US)	6	6
		Japan (JP)^a^	1	11
		Indonesia (ID)^a^	12	14
		Hong Kong (HK)^a^	3	23

*Note*: *N*_ind_ indicates the number of specimens included in the study prior any data filtering.

^a^ These specimens were included at the data analysis stage and are already published ddRAD data (Schmidt, Chung, Honnen, et al., 2020).

We used different sets of samples to address different questions. The *1.native_invasive* dataset includes the core dataset (*2.europe*, see below), plus additional samples collected outside of the target study area to facilitate detection of potential long‐range introductions and their origins. The dataset comprises a total of 208 specimens, including 5 populations from the USA and Brazil, as they are considered to be a bridgehead for the European invasion (Battaglia et al., 2016), and 5 populations from the native range, Japan (Matsuyama), Indonesia (Bandung), and China (Hong Kong) (Schmidt, Chung, Honnen, et al., 2020), representing the three major genetic clusters previously detected in this species native range (Kotsakiozi et al., 2017; Sherpa et al., 2019). Table 1 details the new samples analyzed for this study and the ones with existing ddRAD data obtained from another already published study (Schmidt, Chung, Honnen, et al., 2020).

The samples collected specifically for this study constitute the core dataset (*2.europe*) and were collected during summer months in 2006, 2016, 2017, and 2018. This dataset includes samples from across the Alps in Switzerland, neighboring countries (Germany, France, Liechtenstein, and Italy), and from Albania and Greece. Samples from across the Alps included collections made along national highways around and in the city of Basel in Northern Switzerland (Figure 1c, CH‐North) and collections made in Ticino in southern Switzerland (Figure 1c, CH‐South). To allow assessment of overwintering ability and the presence of self‐sustainable populations, we also included collections made at multiple time points (2017 and 2018) from four locations across the Alps: Strasbourg in France, and Mendrisio, Luzern, and Basel in Switzerland. Samples from neighboring countries included Southern Germany (Baden‐Württemberg), eastern France (Haut‐Rhin), Liechtenstein, and 15 locations across Italy. We sampled Italy more comprehensively than the other neighboring countries because it is considered the most likely source of introduction into Switzerland (Sherpa et al., 2019, Figure 1b). We also included collections from Albania and Greece to provide larger geographic context and to include the region where *Ae. albopictus* was first reported in Europe (Adhami & Reiter, 1998).

The 160 specimens that did not have published ddRAD data available (see Table 1 for details on new vs. already published data) were collected as adults for DNA extraction of the full body or were collected as eggs or larvae and reared to the adult stage before DNA extraction. Larvae were caught by dipping into standing water and eggs were collected with ovitraps (for trap design, see Flacio et al., 2016). Larvae and eggs were reared to adults in a HPP110 constant climate chamber (Memmert GmbH + Co. KG, Schwabach, Germany), mimicking summer temperature and humidity regimes in southern Switzerland. To avoid sampling of siblings, one individual per dip/ovitrap was used. Adults were collected in Biogents Sentinel version 1 traps (Biogents AG, Regensburg, Germany), sent to us through citizen reports, or incidentally caught by the authors as human landing catches. Upon collection, samples were stored in 80% ethanol at 4°C until further processing.

In all analyses, mosquitoes collected from within the same city and in the same year were considered to be from one population. The minimum and maximum distances of samples within the same city are reported in the Appendix S1 (Table S1). This grouping of mosquitoes into populations for analysis is supported by previous estimates of *Ae. albopictus* dispersal that indicate highly localized and restricted active dispersal distances within urban areas (Vavassori et al., 2019). There is evidence that the sample sizes used are adequate because previous studies indicated that with >1000 SNPs, as few as two individuals per population provide adequate resolution to assess genetic differentiation and evolutionary relationships (Kotsakiozi et al., 2017; Nazareno et al., 2017; Willing et al., 2012). In our study, for some locations, only one individual was available (Table 1).

### 2.2 DNA extraction and ddRAD library construction

We extracted total genomic DNA from 160 individual mosquitoes—137 specimens in the core dataset (*2.europe*), as well as 23 specimens in the global dataset (*1.native_invasive)* that did not have published ddRAD data available (6 specimens from USA, 17 from Brazil, see Table 1 for details on new vs. already published data). DNA was extracted from adult mosquito specimens, using the High Pure PCR Template Preparation Kit (Roche, Rotkreuz, Switzerland), following the manufacturer's protocol with an additional step of RNAse treatment. DNA amounts were quantified with a Qubit 2.0 Fluorometer (Invitrogen, Carlsbad, CA, USA).

We constructed the ddRAD libraries following the protocol for *Ae. albopictus* described in Schmidt, Chung, Honnen, et al. (2020) and Schmidt, Chung, Van Rooyen, et al. (2020), an adaptation of the original protocol of Rašić et al. (2014). DNA was digested with the restriction enzymes NlaIII and MluCI (New England Biolabs, Beverly MA, USA). The size selection step targeted a fragment size between 350 and 450 bp. We allocated individuals from the same collection sites randomly across libraries (O'Leary et al., 2018).

We sequenced these same 160 individual mosquitoes—with pools of barcoded DNA of 56 specimens per library on an Illumina HiSeq 2500 system (Illumina Inc., San Diego, CA, USA) at the Department of Biosystems Science and Engineering (D‐BSSE), ETH Basel, Switzerland, using paired‐end, HiSeq Flow Cell v4, HiSeq SBS Kit v4, and with a 10% PHiX spike.

### 2.3 Data processing and SNP genotyping

We used the *process_radtags* function in STACKS v2.2 (Catchen et al., 2013) to de‐multiplex the raw reads and mapped them to the *Ae. albopictus* reference genome (Accession number: GCA_006516635.1) available on NCBI GenBank (Palatini et al., 2020) using the BWA‐MEM algorithm implemented in the Burrows‐Wheeler Aligner tool BWA v0.7.17 (Li & Durbin, 2009), allowing up to four mismatches. For SNP calling, we used the *ref_map.pl* wrapper in STACKS. The VCF file output was used to filter the data for sequencing and SNP call quality. Using VCFtools v1.9 (Danecek et al., 2011) and R version 4.0.3 (R Core Team, 2020), we excluded loci that mapped to repetitive regions of the genome, had more than 50% missing data, or did not exhibit allele balance. We included only bi‐allelic variants, with a maximum mean depth value of 30 and with a minimum allele count of three.

We used plink v1.9 (Chang et al., 2015) to include only individuals with less than 20% missing genotypes and a genotyping rate greater than 80% in iterative steps for the *1.native_invasive* and *2.europe* datasets, independently. We excluded tags with more than 10 SNPs and used the *populations* function in STACKS to obtain output files in VCF format. Since most of the downstream analyses require that SNPs are unlinked, we removed linked sites by excluding SNPs located within a window of 400 bp (i.e., option *‐‐thin* 400) with VCFtools. The window size corresponded to our maximum fragment size, thus each SNP belongs to a single DNA fragment. After conducting a relatedness analysis, we excluded one individual per sibling pair from the analyses (see section below). The reduced dataset was split into two cleaned datasets: *1.native_invasive_cleaned*, including 153 samples and 4714 loci and SNPs, and *2.europe_cleaned*, including 93 samples and 6308 loci and SNPs (Table 3).

### 2.4 Relatedness analysis

To exclude closely related individuals that could potentially bias the analysis of population structure, we calculated Loiselle's *k* (Loiselle et al., 1995), using the program SPAGeDi (Hardy & Vekemans, 2002) for the datasets *1.native_invasive* and *2.europe*. We identified putative full siblings based on pairwise *k* values of >0.1875, and putative half‐siblings with values ranging from 0.1875 > *k* > 0.0938, following Iacchei et al. (2013). The same cutoff values have also been used in a previous study on mosquitoes (Schmidt et al., 2018). In addition to SPAGeDi, we confirmed the putative relationships between individuals with two additional approaches. First, we confirmed relatedness analysis with the *‐‐relatedness2* flag of VCFtools (Danecek et al., 2011) based on the KING inference (Manichaikul et al., 2010) and selected only pairs of siblings identified by both SPAGeDI and VCFtools. Second, we used the software program ML‐Relate (Kalinowski et al., 2006) to confirm putative relationships as described in Schmidt et al. (2018). We run two specific hypotheses of putative relationships: we ran a first “standard” test assuming that the kinship category assigned using Loiselle's k was more likely than the next most likely kinship category. Second, we run a “conservative” test that assumed that the kinship category assigned using Loiselle's k was less likely to be correct. Thus, for pairs with *k* > 0.1875, statistical tests run with ML‐Relate would determine whether the identified pair was full siblings or half‐siblings, while for pairs with 0.1875 > *k* > 0.09375, tests would help determine whether the identified pair was full siblings, half‐siblings, or unrelated. Conservative and standard tests were run using 10,000 simulations of random genotype pairs.

### 2.5 Genetic structure

To assess population structure, we employed both model‐free and model‐based approaches. First, we employed the model‐free Principal Components Analysis (PCA) and Discriminant Analysis of Principal Components (DAPC) (Jombart et al., 2010) on the *1.native_invasive_cleaned* and *2.europe_cleaned* dataset, using the *adegenet* v2.0 package in R (Jombart & Ahmed, 2011). PCA is a multivariate analysis used to identify genetic clusters, without an assumption about the underlying population genetics model. The DAPC analysis maximizes the between group while minimizing the within‐group variance and computes a PCA, followed by a discriminant analysis to identify the number of genetic clusters (Jombart et al., 2010). We used the function *find.clusters* to estimate the number of clusters *K* and *xvalDapc* option to perform cross‐validation and to assess the most likely number of principal components to retain in the DAPC analysis. We used two model‐based approaches. The first approach was the maximum‐likelihood method implemented in the program ADMIXTURE v1.3.0 (Alexander et al., 2009). We used it to conduct ancestry analysis and to estimate the most likely number of evolutionary clusters *K* on the cleaned datasets (*1.native_invasive_cleaned* and *2.europe_cleaned*). The second approach was to choose the most likely value for *K* using the ADMIXTURE's cross‐validation procedure. Genetic differentiation was further investigated with fineRADstructure v1.7.20 (Malinsky et al., 2018). This method enables fine‐scale population structure inference by using a Bayesian clustering approach and it has been shown to be especially informative in the case of recent gene flow between mosquito populations (Pichler et al., 2019). For this analysis, we only used the *1.native_invasive* dataset because the algorithm takes into account haplotype information and uses all available SNPs allowing for a higher structural resolution (Malinsky et al., 2018).

### 2.6 Genetic differentiation, isolation by distance, and overwintering

To evaluate the degree of genetic differentiation, we estimated pairwise *F*_st_ values (Weir & Cockerham, 1984) at country level on the dataset *1.native_invasive_cleaned* with the R package *HierFstat* v0.5–10 (Goudet, 2005) and estimated the corresponding 95% confidence intervals by performing 1000 bootstraps over all loci. Next, we calculated individual inbreeding coefficients (*F*_IS_) by assessing their statistical significance with 1000 bootstrap samplings and estimated allelic richness (AR). We calculated the observed heterozygosity (*H*_*O*_) using VCFtools (*−het*). Individuals were grouped by country and differences in mean *H*_*O*_ between groups were tested with a non‐parametric Kruskal–Wallis (KW) test for statistical significances in R. Expected heterozygosity (*H*_*E*_) per country was computed using the R package *adegenet* v2.0.

To investigate patterns of genetic diversity, genotype frequency, and genetic differentiation across Europe (*2.europe_cleaned* dataset), we estimated *F*_ST_, *F*_IS_, AR, *H*_*O*_, and *H*_*E*_ using the R package *adegenet* v2.0, *F*_IS_ and pairwise *F*_ST_ using the R package *HierFstat* (Goudet, 2005) and pairwise proportion of shared alleles (*D*_ps_) using the R package *adegenet* (propShared function). We visualized pairwise *D*_ps_ with neighbor‐net networks with the software SplitsTree v5.0 (Huson & Bryant, 2006). To assess the impact of geographic distance on genetic differentiation, we performed a test for isolation by distance (IBD) with a Mantel test (Mantel, 1967) with 1000 permutations, using *D*_ps_ and log‐transformed geographic distances as the input, *r* = 0 as the null hypothesis, and *r* > 0 as the alternative hypothesis. We used pairwise *D*_ps_ rather than *F*_ST_ in the test for IBD because this metric provides improved power to detect IBD at small geographical scales, with small genetic distances as expected due to the recent invasion history, high dispersal, and small sample sizes (Bowcock et al., 1994) (Shirk et al., 2017), which is characteristic for the *2.europe_cleaned* dataset.

To assess overwintering ability and assess the presence of self‐sustainable populations, we estimated pairwise *F*_ST_ values between individuals from Mendrisio, Luzern, and Basel that had been collected during two consecutive mosquito seasons in 2017 and 2018. To evaluate if *Ae. albopictus* overwinters in our study area, we compared the *F*_ST_ values from the temporal comparison with the *F*_ST_ values calculated among individuals from geographically distant locations. If overwintering does occur, the samples collected in the two different years would belong to the same population and, therefore, their *F*_ST_ values should be considerably smaller than across geographically distant populations.

### 2.7 Genetic assignment test

In order to identify possible source population of the mosquitoes in Switzerland, we performed genetic assignment test with the R program *assignPOP* (Chen et al., 2018). We assigned individuals collected in France and in Northern Italy as source populations, considering their geographical proximity to Switzerland. We tested assignment accuracies via Monte Carlo cross‐validation based on the following parameters: proportion of individuals used in training set: 0.5, 0.7, and 0.9; proportion of loci used in training set: 0.25,0.5, and 1 and loci sample method F_ST_; iterations: 30; and model: support vector machine.

## 3 RESULTS

### 3.1 SNP discovery

We sequenced 160 individuals obtaining a total of 828 million reads, with 5 million reads per sample on average, ranging from 7 thousand to 17 million. After filtering and removal of duplicate siblings, the dataset *1.native_invasive_cleaned* included 153 individuals and 4714 SNPs and loci. The dataset *2.europe_cleaned* included 93 samples and 6308 SNPs. An average of 3 million reads (73%) per individual aligned to the reference genome. Table 3 shows the details on the number of reads, individuals, SNPs, coverage, and level of missing data of each dataset.

### 3.2 Relatedness analysis

We identified 15 full‐ and 10 half‐sibling pairs from the same collection sites (Table 4). One sibling from each pair was kept for all downstream analysis (i.e., *1.native_invasive_cleaned* and *2.europe_cleaned*). Although we had only 15 full siblings in our dataset, we found two siblings from the same location in two consecutive years. These were detected at a distance of 330 m in 2017 and 2018 in Strasbourg, France. The first sibling was collected from an egg sample in fall 2017, while the second one was caught as an adult during the second half of July 2018.

### 3.3 Genetic structure

**FIGURE 2:**
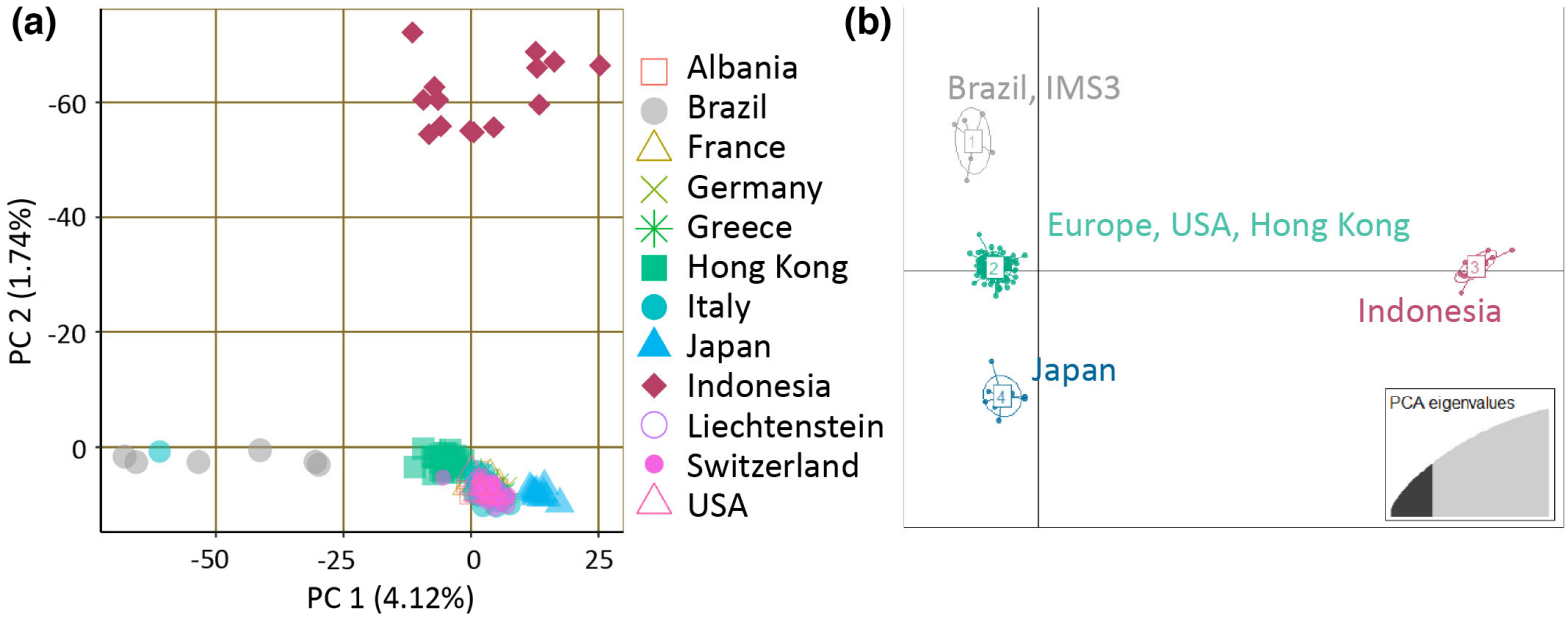
Genetically distinct clusters of *Ae. albopictus* sampled populations. Four genetic clusters may be observed in the global dataset (*1.native_invasive_cleaned*) based on the PCA and DAPC analysis. (a) PCA with the percent variation explained by the first two principal components (b) Scatterplot showing the results from the DAPC (*K* = 4).

**FIGURE 3:**
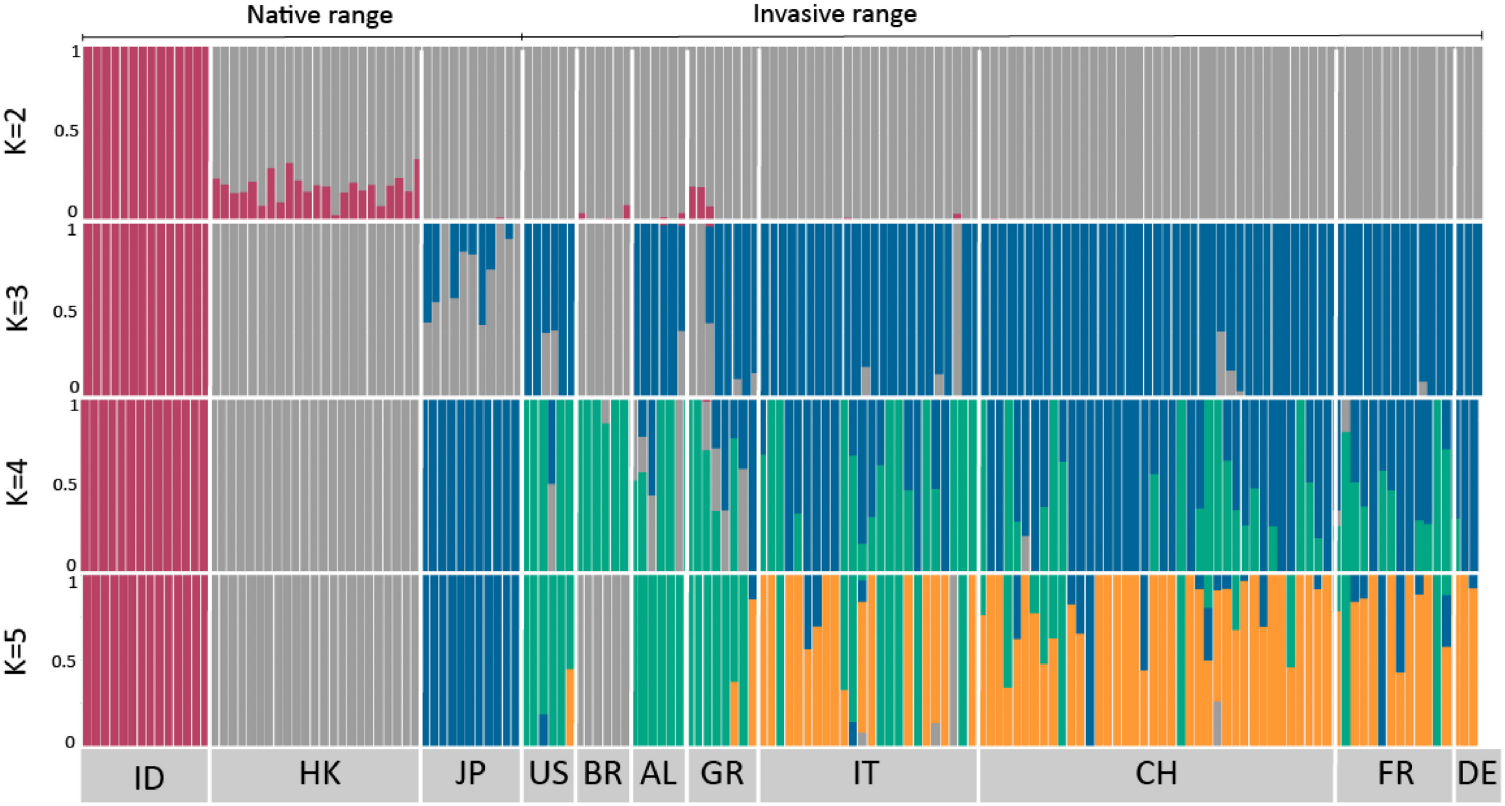
*Aedes albopictus* ADMIXTURE barplot for all mosquito populations based on the results from the dataset *1.native_invasive_cleaned*. Each bar represents one individual, while white vertical lines indicate separate countries. Country codes: AL: Albania, AU: Austria, BR: Brazil, CH: Switzerland, HK: Hong Kong, DE: Germany, FR: France, GR: Greece, ID: Indonesia, FL: Liechtenstein, IT: Italy, JP: Japan and US: USA.

**FIGURE 4:**
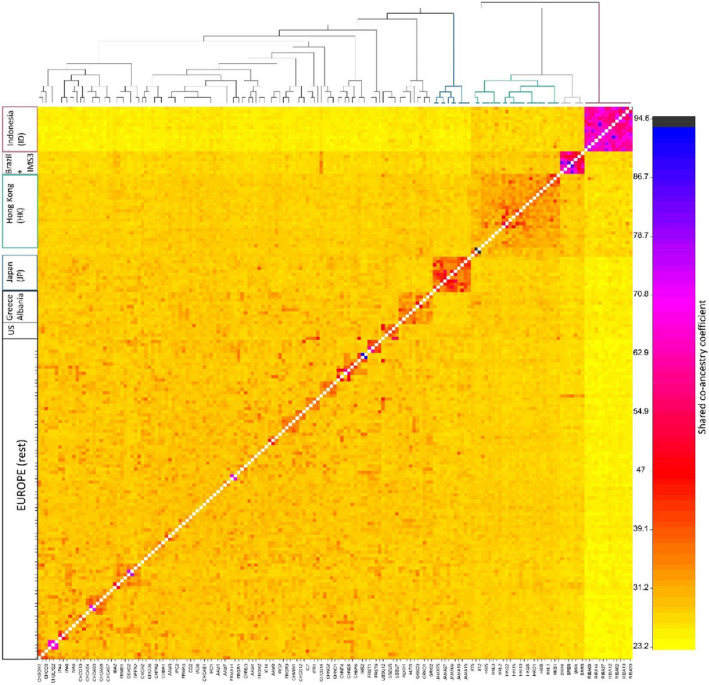
Output of the fineRADstructure analysis of the *1.native_invasive* dataset. The heat map indicates pairwise co‐ancestry between individuals, with black, blue, and purple representing the highest levels, red and orange indicating intermediate levels, and yellow representing the lowest levels of shared co‐ancestry. The tree on top of the heat map shows the inferred relationships between the specimens analyzed, with each tip corresponding to an individual. On the Y‐axis, country of origin with their sample collection site ID is reported if they create distinct clusters, otherwise are included in the Europe (rest) cluster. On the X‐axis, sample codes are encoded with their laboratory ID (see Appendix S1: Table S1). Siblings are depicted in black and blue colors.

At the global level, the DAPC analysis separated the specimens in four main clusters (*1.native_invasive_cleaned* dataset Figure 2b, Table 5): cluster 1 (gray) consists of the mosquitoes collected in Brazil and one specimen from Sicily (Sample ID: IMS3, Messina); cluster 2 (green) includes specimens collected in Europe, Hong Kong, and USA; clusters 3 (pink) and 4 (blue) comprise the specimens from Indonesia and Japan, respectively. A similar clustering was also recovered by the PCA analysis (Figure 2a): specimens from Indonesia, Brazil, and Japan were the most differentiated, whereas specimens from Europe, Hong Kong, and USA largely overlapped. The ADMIXTURE cross‐validation analysis supports the presence of two genetic clusters in the *1.native_invasive_cleaned* dataset with individuals collected in Indonesia forming one cluster genetically distinct from all the other individuals (Figure 3 *K* = 2). However, as the cross‐validation errors were very similar for a number of *K*'s (*K* = 1: 0.216, *K* = 2: 0.211, *K* = 3: 0.219, *K* = 4: 0.232, *K* = 5: 0.247), we further explored *K* = 2, 3, 4, and 5. *K* = 4 appears to be the most likely scenario with two clearly distinct genetic groups, including specimens collected in Indonesia and Hong Kong, and two genetic groups with a high level of genetic admixture, including specimens collected in Europe, Brazil, the USA, and Japan (Figure 3). The fineRADstructure analysis confirms the presence of four genetic clusters at the global scale (Figure 4).

**FIGURE 5:**
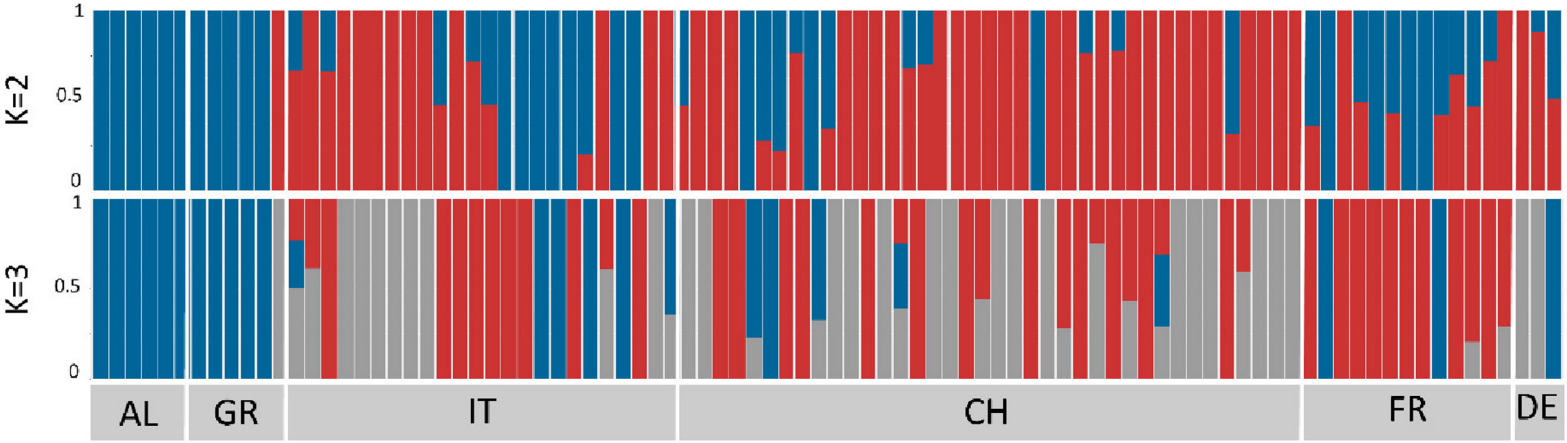
ADMIXTURE barplot obtained for the *2.europe_cleaned* dataset for *K* = 2–3. Individuals represented by vertical bars along the plot grouped by country and collection site. The Y‐axis represents the probability of an individual to be assigned to a genetic cluster. Each cluster is given in a different color. Multi‐colored bars indicate admixed genetic ancestry in the respective individual. The white vertical lines indicate country limits. Country codes: AL: Albania, CH: Switzerland, DE: Germany, FR: France, GR: Greece, IT: Italy.

**FIGURE 6:**
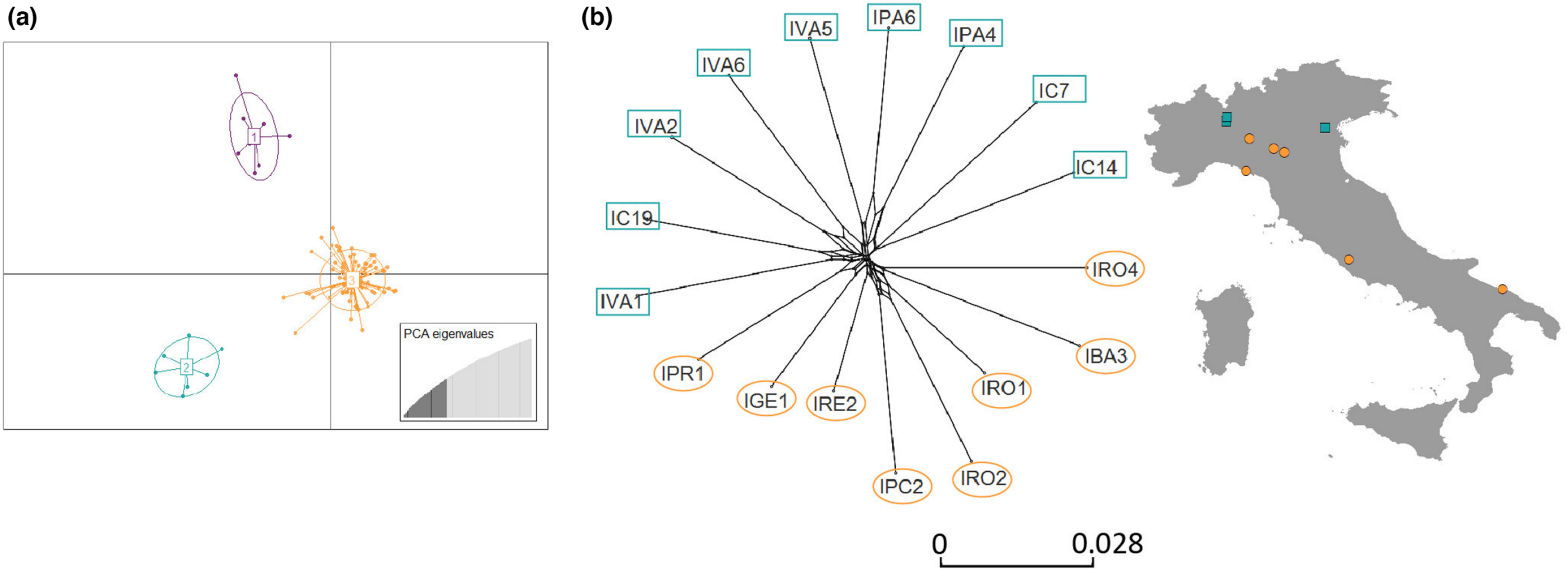
Genetic structure and differentiation of *Ae. albopictus* specimens collected in Europe. (a) Scatterplot showing the results of the DAPC (*K* = 3) on the *2.europe_cleaned* dataset. Cluster 1‐purple includes mosquitoes collected in Albania with some specimens collected in Greece; cluster 2‐green includes mosquitoes collected in Northern Italy (with the exception of two specimens which clustered with cluster 3 (orange), mosquitoes collected in southern and northern Switzerland and one specimen from Germany. Cluster 3‐orange includes specimens collected in Italy‐Center‐South, Italy‐Sicily, Switzerland, and France. (b) Neighbor‐net network of *D*_ps_ relative genetic distances among the specimens from Italy. The map shows the locations of the sampling in the region of Italy‐Center‐South and Italy‐Sicily. Specimens collected in Northern Italy are depicted with a green square (cluster 2 ‐ green) and the one collected in Central and Southern Italy with orange circles (cluster 3 ‐ orange). Specimens from the Italian island Sicily are not reported here. For the sample abbreviations, see Appendix S1, Table S1 Laboratory ID.

At the European level, different analysis suggests a weak substructuring. In the ADMIXTURE analysis, the individuals collected in Europe, except for some individuals from Greece and Albania, shared high levels of genetic ancestry and showed the highest proportion of their genome being assigned to either cluster 2 or 4 (green and blue clusters in Figure 3). Two specimens collected in Albania and Greece, respectively, were also assigned to cluster 1, together with mosquitoes collected in Hong Kong. The results for other values of K (*K* = 2, 3, and 5) are reported in Figure 3. The fineRADstructure and the PCA analysis groups together specimens from Albania and Greece, separating them from the rest of the European samples (Figure 4). While the admixture analysis on the European dataset did not detect clear genetic structuring (i.e., optimal *K* = 1; see Figure 5), the DAPC separates the specimens into three clusters (Figure 6). Cluster 1 (Figure 6a – purple) includes all specimens collected in Albania with two additional specimens from Greece. Cluster 2 (Figure 6a – green) comprises all specimens collected from Northern Italy, except for two individuals from Como and Varese, samples from both southern and northern Switzerland, and one individual from Germany. Cluster 3 (Figure 6a – orange) contains specimens from South and North Italy, Greece, all the specimens from France, and 22 specimens from Switzerland (Table 6).

### 3.4 Genetic differentiation, isolation by distance, and overwintering

The degree of differentiation between countries detected in the dataset *1.native_invasive_cleaned* is low, with pairwise *F*_ST_ values ranging from 0 to 0.21, with lower values between specimens from Italy, Switzerland, and France, and higher values between specimens from Indonesia and Switzerland (Table 7).

Observed (*H*_*O*_) and expected (*H*_*E*_) heterozygosity ranged from 0.041 to 0.055 and from 0.048 to 0.075, respectively. *H*_*O*_, *H*_*E*_, and *F*_IS_ within country for the dataset *1.native_invasive_cleaned* are reported in Table 8. *H*_*0*_ differed among countries (K‐W *H* = 41.9, df = 10, *p*‐value <.001) and between the native and the invasive range (K‐W *H* = 6.3, df = 1, *p*‐value <.05). The highest heterozygosity measured within a country was among the Indonesian specimens (Table 8).

To further investigate the dispersion across the Alps and identify the presence of self‐sustaining populations, we grouped specimens from Italy and Switzerland according to the time since their first report of introduction into “long‐established” (i.e., established since 1990) and “recently‐established” (after 2003) populations. With this approach, we identified three groups of populations from Italy (Figure 1b i.e., IT‐North, IT‐Center‐South, and IT‐Sicily) and two groups of recent population from Switzerland (Figure 1c i.e., CH‐North and CH‐South) and compared their genetic diversity.

**FIGURE 7:**
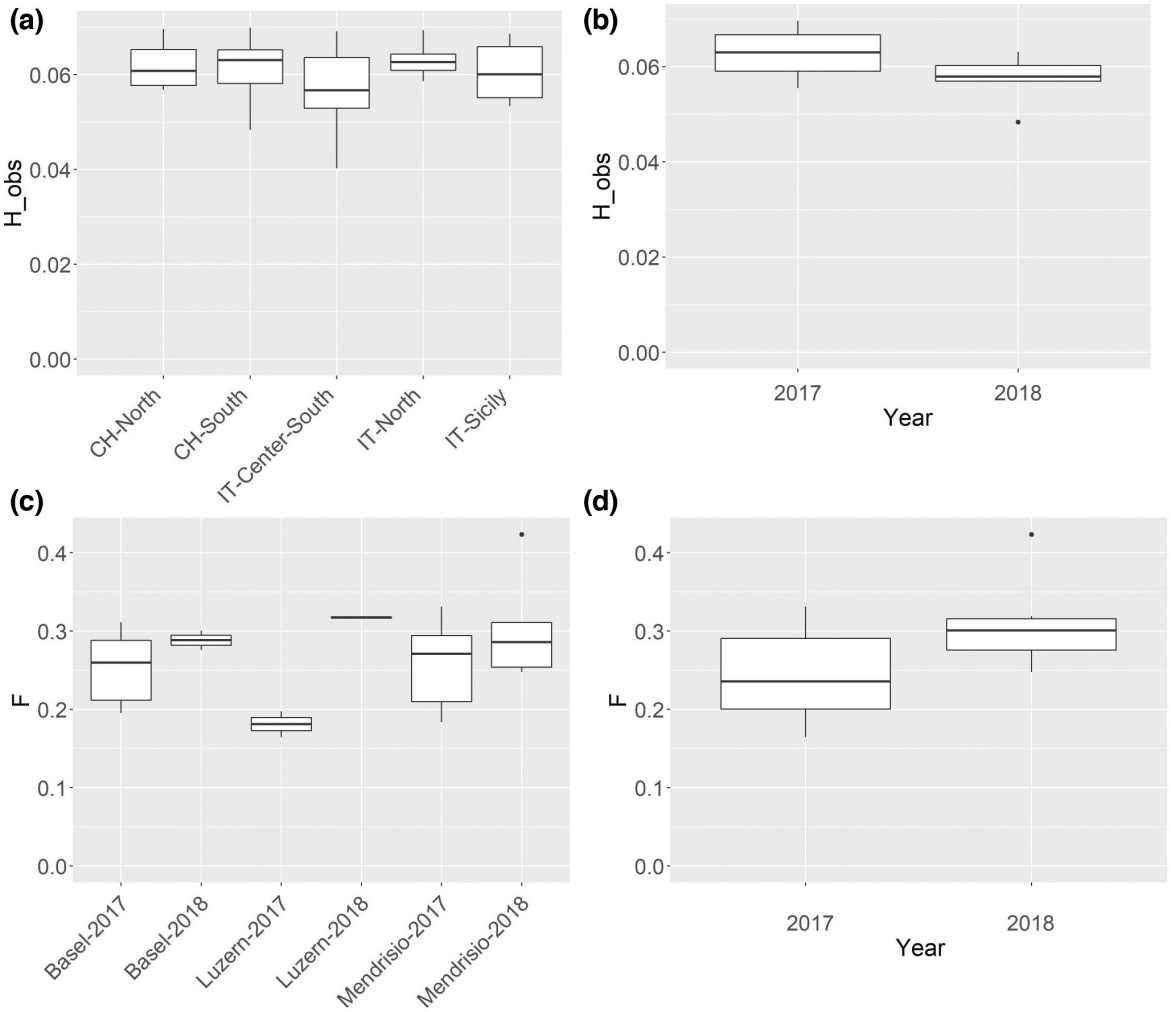
Genomic diversity of *Ae. albopictus* collected in Italy and in Switzerland. (a) Individual observed heterozygosity (H_obs) estimated with VCFtools on the *2.europe_cleaned* dataset. The individuals were grouped by geographical regions, including three regions in Italy and two regions in Switzerland, and difference in their mean heterozygosity (*H*_O_) was tested with the non‐parametric Kruskal–Wallis (KW) test. (b) H_obs in the samples collected in Switzerland from the same sites in 2017 and 2018. (c) *F*_IS_ calculated between samples collected in the three sites in Switzerland in 2017 and in 2018. (d) Inbreeding coefficient *F*_IS_ between specimens collected from the same sites in Switzerland in 2017 and 2018.

The recently established populations in Switzerland (CH‐North and CH‐South) did not show lower genetic diversity (i.e., *H*_*O*_ and *H*_*E*_) than the long‐established Italian populations (IT‐North, IT‐Center‐South, and IT‐Sicily) (K‐W *H* = 2.81, df = 4, *p*‐value = .59; Figure 7a). Pairwise *F*_ST_ between collection sites in Switzerland ranged between 0 and 0.04 (Appendix S1, Table S3). For the Swiss locations, Mendrisio, Luzern, and Basel, we found a decrease of 0.004 in the heterozygosity between the specimens collected in 2018 versus the samples collected in 2017 (K‐W *H* = 4.59, df = 1, *p*‐value <.05; Figure 7b) and higher inbreeding coefficients within the three investigated sites (Figure 7c and d). Pairwise *F*_ST_ values among the specimens collected in the three sites at multiple time points were similar (Mendrisio: 0.006, Luzern: 0, Basel: 0. Appendix S1, Table S3) and considerably smaller than values calculated between geographically distant populations (Appendix S1, Table S3). The specimens from Strasbourg, France, were excluded from this analysis because they were identified as full siblings (see relatedness analysis, Table 4).

**FIGURE 8:**
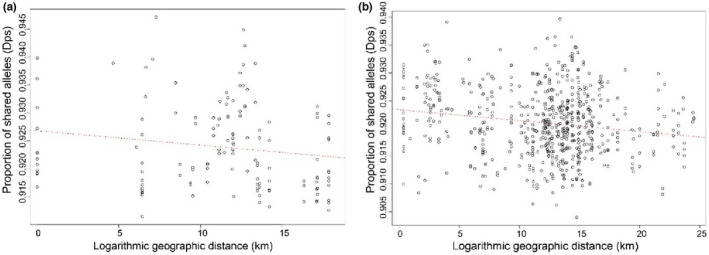
Isolation by distance (IBD) analysis using the *2.europe_cleaned* dataset represented as scatterplots. (a) Correlation of genetic distances as proportion of shared alleles (*D*_ps_) and geographic distances on logarithmic scale for samples from Italy (excluding samples from Sicily). The correlation was assessed using a Mantel test, *R* = −0.16, *p*‐value = .974 based on 999 replicates. (b) Correlation between *D*_ps_ genetic distances and logarithmic geographic distances for samples from Northern Italy and southern Switzerland. The correlation was assessed using a Mantel test based on 999 replicates, Mantel *R* = −0.17, *p*‐value = .988.

Pairwise *D*_ps_ between individuals ranged between 0.88 and 0.93. We did not find any indication of isolation by distance among the samples collected in Italy and Switzerland in the *2.europe_cleaned* dataset (Mantel *R* = −0.17, *p*‐value = .988; Figure 8) even if we only included the samples collected in mainland Italy, excluding samples from Sicily (Mantel *R* = −0.16, *p*‐value = .974; Figure 8). In contrast, the neighbor‐net tree based on *D*_ps_ distances shows a separation between samples from Northern and Southern Italy (Figure 6b).

### 3.5 Genetic assignment test

**FIGURE 9:**
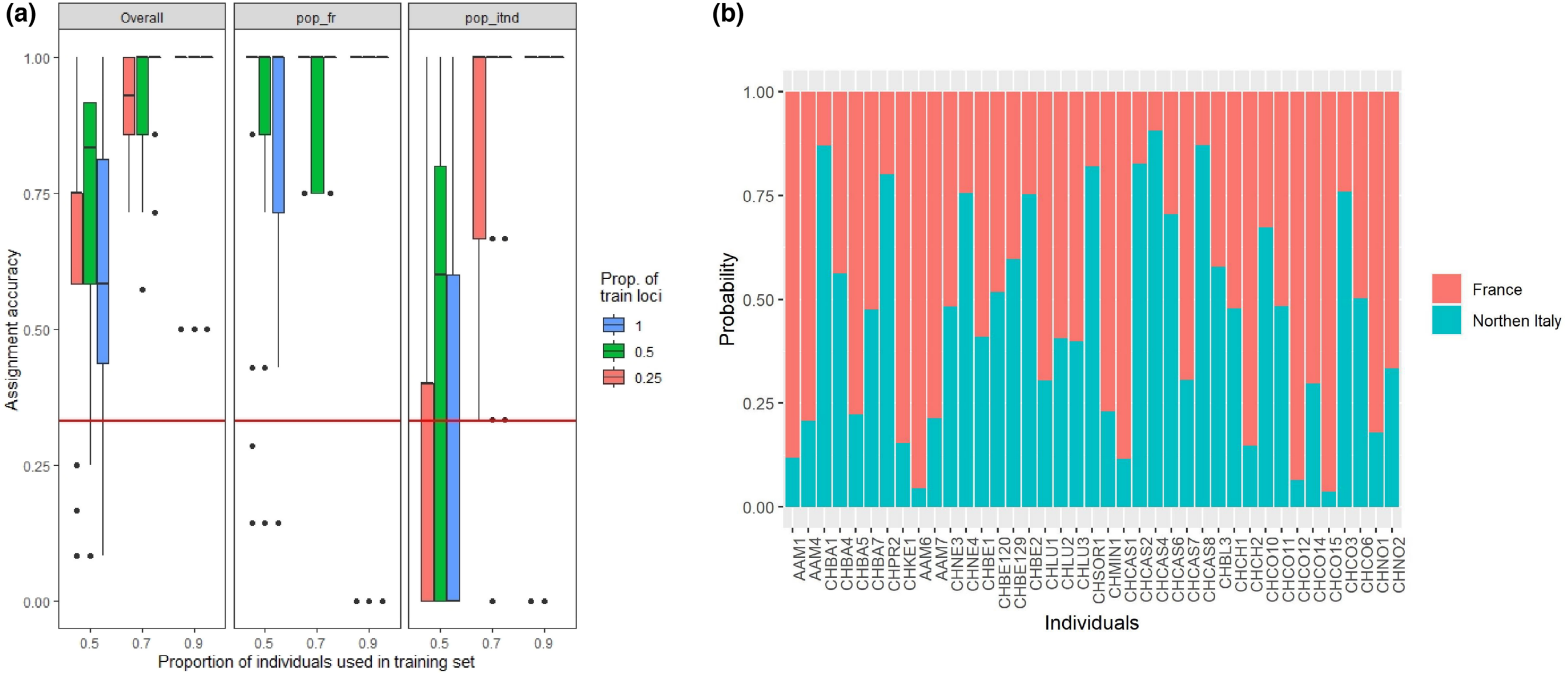
Genetic assignment tests. (a) Assignment accuracy estimated by Monte Carlo cross‐validation based on the *2. europe_cleaned* dataset. Assigned source populations were France (pop_fr) and Northern Italy (pop_itnd). Red horizontal lines indicate 0.33 null assignment rate, where the assignment accuracy is zero. (b) Membership probability of the individuals collected in Switzerland, organized from north to south. Individuals are sorted based on the probability of assignment to their original populations.

We performed genetic assignment tests on individuals from population collected in Switzerland, using the method implemented in assignPOP. Due to their geographical proximity, individuals collected in France and in Northern Italy were assigned as source populations. Assignment accuracies of individuals collected in Italy (pop_itnd) are relatively low, whereas those collected in France (pop_fr) are higher (Figure 9a). Simulations performed best when all loci and individuals were used. On average, 41% of the individuals collected in Switzerland were assigned to Northern Italy and 59% to France, but only 56% of individuals were assigned with a proportion of genetic constitution of >75%, which can be considered as effective assignment (Figure 9b).

## 4 DISCUSSION

Our aim was to describe the invasion history of *Ae. albopictus* into Switzerland and across the Alps, and to estimate if the current populations are self‐sustaining. As a point of reference, we compared the genetic variability in mosquitoes from Switzerland to populations from Italy that have been established for over 25 years, to recently established populations in neighboring France, Germany, and Liechtenstein, and to populations from the mosquito's native range in Japan, Indonesia, and China. We found that populations from Switzerland had similar genetic variability to those from well‐established populations in Italy (Figure 7a, Table 2), and that there were no clear patterns of isolation by distance (Figure 8). We detected weak genetic structuring with a high level of genetic admixture, supporting a scenario of rapid expansion after introduction into Switzerland—both south and north of the Alps (Figures 2, 3, 4). These findings are in line with observations from the Swiss national monitoring program, suggesting human‐aided dispersal along main transportation routes (Müller et al., 2020). While the genetic pattern suggests frequent re‐introductions from Italian sources, the recovery of a pair of full siblings at a distance of 330 m in Strasbourg (France) in two consecutive years (Table 4) suggests the presence of an overwintering population north of the Alps. To our knowledge, this result is an indirect molecular evidence for establishment of a self‐sustaining population north of the Alps.

**TABLE 2:** Basic diversity statistics for the dataset *2.europe_cleaned*

Geographical regions	*N*_ind_	Private alleles	Mean *H*_O_	Mean *H*_E_	AR	*F*_IS_	*F*_is_ CI	
							Upper	Lower
CH‐North	12	133	0.062	0.080	1.085	0.269	0.255	0.297
CH‐South	27	447	0.062	0.085	1.087	0.249	0.247	0.274
Italy‐North	9	122	0.063	0.082	1.088	0.241	0.257	0.298
Italy‐Center‐South	8	92	0.057	0.07	1.077	0.244	0.208	0.257
Italy‐Sicily	6	65	0.061	0.072	1.081	0.254	0.211	0.270
France	14	195	0.058	0.073	1.078	0.299	0.224	0.260
Germany	3	33	0.064	0.071	1.086	0.227	0.256	0.327
Albania	6	105	0.058	0.073	1.082	0.294	0.258	0.309
Greece	6	107	0.062	0.075	1.083	0.225	0.204	0.252

Abbreviations: *N*_ind_, number of individuals; Mean *H*_*O*_, mean observed heterozygosity; Mean *H*_*E*_, mean expected heterozygosity; AR, allelic richness; *F*_IS_, inbreeding coefficient with 95% confidence interval (*F*_IS_ CI).

**TABLE 3:** Details on the datasets used in the study

Dataset	*N*_ind_	Loci (*N*)	SNPs (*N*)	Missing data (samples ± SD)	Missing data (locus ± SD)	Average read depth per individual ± SD	Average coverage per site ± SD
*1.native_invasive*	208	4930	23,240	11.1% ± 3.9	11.1% ± 5.8	12.9 ± 7.5	12.9 ± 6.6
*1.native_invasive_cleaned*	153	4714	4714	11.1% ± 4.0	11.1% ± 5.9	12.9 ± 7.3	13.1 ± 6.8
*2.europe*	137	9966	9960	12.18% ± 3.7	12.17% ± 6.0	13.1 ± 6.5	13.2 ± 6.7
*2.europe_cleaned*	93	6308	6308	10.6% ± 3.3	10.6% ± 6	12.2 ± 6.5	13.9 ± 6.6

**TABLE 4:** List of individuals and their relative kinship identified in the *1.native_invasive* and *2.europe* dataset in the relatedness analysis

Individual ID 1	Individual ID 2	Loiselle k (SPAGeDi)	Kinship (VCFtools)	Kinship (ML‐Relate)
CHCAS2	CHCAS3	0.513879 (FS)	FS	FS
CHBE121	CHBE122	0.512615 (FS)	FS	FS
DEHD2	DEHD3	0.509926 (FS)	FS	FS
CHPR1	CHPR2	0.492123 (FS)	FS	FS
FRMA11	FRMA9	0.473704 (FS)	FS	FS
CHBE121	CHBE129	0.471598 (FS)	FS	FS
CHBE122	CHBE129	0.470286 (FS)	FS	FS
CHNE1	CHNE5	0.453771 (FS)	FS	FS
FRST10	FRST1	0.445381 (FS)	FS	FS
CHNE2	CHNE5	0.325529 (FS)	FS	FS
IPA4	IPA5	0.318006 (FS)	FS	FS
AAM6	AAM8	0.312713 (FS)	FS	FS
CHNE2	CHNE4	0.306556 (FS)	FS	FS
IBA2	IBA3	0.301730 (FS)	FS	FS
CHNE4	CHNE5	0.286318 (FS)	FS	FS
FRST1	FRST4	0.194805 (FS)	HS	HS
FRST4	FRST9	0.188862 (FS)	HS	HS
FRST10	FRST4	0.179891 (HS)	HS	HS
FRST1	FRST9	0.165271 (HS)	HS	HS
CHBA6	FRSL1	0.162914 (HS)	HS	HS
FRST4	FRST5	0.159820 (HS)	HS	HS
AAM3	AAM4	0.148871 (HS)	HS	HS
FRCO3	FRCO4	0.145682 (HS)	HS	HS
CHNE1	CHNE2	0.136978 (HS)	HS	HS
FRCO2	FRCO4	0.120723 (HS)	HS	HS
FRST10	FRST5	0.090178 (UR)	UR	UR
CHCO11	CHCO15	0.088116 (UR)	UR	UR
CHCAS1	CHCAS6	0.084865 (UR)	UR	UR
FRGR7	FRGR9	0.083812 (UR)	UR	UR
FRGR3	FRGR8	0.082819 (UR)	UR	UR
IBA3	IBA5	0.079113 (UR)	UR	UR
CHLU2	CHLU3	0.071667 (UR)	UR	UR
CHCAS3	CHCAS8	0.065919 (UR)	UR	UR
CHCO14	CHCO6	0.062289 (UR)	UR	UR
IC14	IC7	0.061200 (UR)	UR	UR
FRCO2	FRCO3	0.060119 (UR)	UR	UR
FRGR2	FRGR8	0.056716 (UR)	UR	UR
IVA2	IVA6	0.055244 (UR)	UR	UR

*Note*: Kinship was determined by three different methods, first using SPAGeDi where pairs of *k* > 0.1875 are identified as full siblings (FS), those of 0.0938 < *k* < 0.1875 as half‐siblings (HS) and those *k* < 0.0938 as unrelated individuals (UR), second using VCFtools (flag ‐relatedness2) based on a relationship inference algorithm and lastly ML‐Relate to run specific hypothesis tests of putative relationships assigned by SPAGeDi. We added to this table the values of 14 randomly selected individuals from the same population and their respective Loiselle K values. Sample information for each individual ID listed is reported in the Appendix S1.

**TABLE 5:** Composition of the DAPC groups obtained for the *1.native_invasive_cleaned* dataset (Figure 2b)

Country	Cluster 1	Cluster 2	Cluster 3	Cluster 4
Indonesia (ID)	0	0	14	0
Hong Kong (HK)	0	23	0	0
Japan (JA)	0	0	0	11
USA (US)	0	6	0	0
Brazil (BR)	6	0	0	0
Albania (AL)	0	6	0	0
Greece (GR)	0	8	0	0
Italy (IT)	1	22	0	0
Switzerland (CH)	0	39	0	0
France (FR)	0	13	0	0
Germany (DE)	0	3	0	0
Liechtenstein (FL)	0	1	0	0

**TABLE 6:** Composition of the DAPC groups obtained for the *2.europe_cleaned* dataset (Figure 6a)

Country	Cluster 1	Cluster 2	Cluster 3
Albania	5	0	1
Greece	2	1	3
Italy	0	6	16
Switzerland	0	12	27
France	0	0	13
Germany	0	2	1
Liechtenstein	0	0	1

**TABLE 7:** Pairwise *F*_ST_ values for the dataset *1.native_invasive_cleaned* and the corresponding 95% confidence interval using 1000 bootstrapping in the top diagonal. Pairwise *F*_ST_ was calculated only for populations with *N*_ind_ ≥ 2

	AL	BR	FR	DE	GR	IT	CH	US	HK	JA	ID
AL	NA	0.10–0.14	0.03–0.05	−0.01–0.03	0.01–0.03	0.02–0.04	0.03–0.05	0.05–0.07	0.04–0.07	0.07–0.10	0.16–0.20
BR	0.12	NA	0.10–0.14	0.07–0.11	0.09–0.12	0.08–0.12	0.09–0.13	0.10–0.14	0.07–0.11	0.12–0.15	0.16–0.20
FR	0.04	0.12	NA	0.01–0.04	0.01–0.02	0.00–0.01	0.00–0.01	0.04–0.06	0.03–0.04	0.05–0.07	0.17–0.22
DE	0.01	0.09	0.02	NA	0.00–0.03	−0.01–0.02	0.00–0.03	0.02–0.05	0.02–0.05	0.03–0.06	0.12–0.16
GR	0.02	0.10	0.02	0.02	NA	0.01–0.02	0.01–0.02	0.04–0.06	0.02–0.04	0.06–0.08	0.15–0.19
IT	0.03	0.10	0.00	0.01	0.01	NA	0.00–0.00	0.03–0.05	0.03–0.04	0.05–0.07	0.18–0.22
CH	0.04	0.11	0.00	0.01	0.01	0.00	NA	0.03–0.05	0.03–0.04	0.05–0.07	0.19–0.24
US	0.06	0.12	0.05	0.04	0.05	0.04	0.04	NA	0.04–0.06	0.06–0.08	0.16–0.20
HK	0.05	0.09	0.03	0.04	0.03	0.03	0.04	0.05	NA	0.07–0.09	0.16–0.20
JA	0.08	0.13	0.06	0.04	0.07	0.06	0.06	0.07	0.08	NA	0.19–0.23
ID	0.18	0.18	0.19	0.14	0.17	0.20	0.21	0.18	0.18	0.20	NA

Abbreviations: AL, Albania; BR, Brazil; FR, France; DE, Germany; GR, Greece; IT, Italy; CH, Switzerland; US, USA; HK, Hong Kong; JA, Japan; ID, Indonesia.

**TABLE 8:** Basic diversity statistics for the dataset *1.native_invasive_cleaned* calculated at country level (*N*_ind_ = 153, *N*_snps_ = 4714)

Country	*N*_ind_	Private allele	Mean H_obs	Mean H_exp	*F*_IS_	*F*_IS_ CI (0.025, 0.975)	
Indonesia (ID)	14	643	0.055	0.075	0.288	0.227	0.339
Hong Kong (HK)	23	220	0.044	0.053	0.188	0.148	0.232
Japan (JA)	11	85	0.049	0.060	0.197	0.159	0.253
USA (US)	6	29	0.046	0.052	0.176	0.135	0.244
Brazil (BR)	6	87	0.041	0.051	0.212	0.169	0.293
Albania (AL)	6	15	0.046	0.051	0.116	0.116	0.220
Greece (GR)	8	25	0.046	0.050	0.125	0.009	0.179
Italy (IT)	23	74	0.045	0.054	1.166	0.127	0.205
Switzerland (CH)	39	117	0.046	0.055	0.156	0.132	0.190
France (FR)	13	30	0.042	0.048	0.131	0.096	0.181
Germany (DE)	3	11	0.049	0.049	0.138	0.113	0.237
Liechtenstein (FL)	1	1	–	–	–	–	–

*Note*: *N*_ind_, number of individuals; H_exp, expected heterozygosity; H_obs, Individual observed heterozygosity averaged per geographical regions; *F*_IS_, inbreeding coefficient with 95% confidence interval (*F*_IS_ CI).

Across all of our specimens (within the *1.native_invasive_cleaned* dataset), we detected the presence of four genetic clusters (Figures 2, 3, 4). High levels of shared ancestry were recorded between mosquitoes collected in France, Italy, Switzerland, Germany, and the USA, while the mosquitoes collected in Albania and Greece were genetically distinct from the rest of Europe (Figures 3 and 4). These results suggest that mainland Europe could have been invaded by mosquitoes originating via the USA to Italy as previously proposed (Battaglia et al., 2016; Sherpa et al., 2019; Zhong et al., 2013). While Albania was the first European country invaded by *Ae. albopictus*, our results suggest that samples collected in Albania are genetically closer to samples from Greece and the USA (Figure 3 *K* = 5 and Figure 4). Nevertheless, we may not completely rule out recent gene flow from Albania, while the genetic pattern as well as the geographical isolation of the country in the past rather supports the hypothesis that the invasion on mainland Europe goes back to an origin in the USA. Assigning the primary source with absolute rigor is very challenging considering the very recent colonization of this species in the study area. Our genetic assignment tests aiming to identify primary sources do not reveal the full picture (Figure 9) and, therefore, future studies should consider a denser sampling scheme across Italy, especially the northern regions. In addition to denser sampling, using whole genomes or a larger number of SNPs could help shedding more light on some of the recent invasion histories.

The approaches used to test genetic clustering in our European dataset did not yield entirely consistent results (Figures 5 and 6), suggesting that in Europe, there are at least three different clusters, with some genetic admixture between two of these clusters including specimens from Italy and Switzerland. This finding differs from previous studies (Pichler et al., 2019; Sherpa et al., 2019) that suggested two distinct genetic clusters in Italy, one comprising specimens from Northern Italy originating from the USA, and another one consisting of specimens from the central and southern areas that originated from admixture between the northern Italian genetic cluster and individuals from China. In our data, we also identified one mosquito from Sicily (Messina) that clustered together with mosquitoes collected in Brazil (Figure 2). Previous studies have suggested that the Brazilian populations are genetically distinct from the North American ones (Birungi & Munstermann, 2002) and are only partially related to the ones from the native range. An explanation for this apparent discrepancy might be undersampling of the native range (Kotsakiozi et al., 2017; Pichler et al., 2019), suggesting that the lineage that became invasive in Brazil has not yet been sampled in the native range. The Messina individual might have originated from the same yet unknown native lineage that also invaded Brazil, highlighting the need for future studies.

Overall, there is a genetic similarity of the mosquitoes collected in Switzerland to the one collected in Italy (Figure 3). This similarity, together with the close proximity of the two countries, the intense traffic of goods and people, and surveillance data and lack of isolation‐by‐distance, supports the hypothesis of the introduction of *Ae. albopictus* from Italy to Switzerland. This is in line with previously published studies indicating Italy as the main source for the European spread (Kraemer et al., 2019; Sherpa et al., 2019).

Interestingly, ddRAD‐seq SNP‐based studies with similar genomic resolution on a closely related species, *Ae. aegypti*, found strong spatial genetic structure at even small spatial scales (<4 km in Schmidt et al., 2018 and <200 m in Jasper et al., 2019), suggesting that the weak genetic structuring found in this study is not a result of low genomic resolution. This difference is more likely caused by different dispersal abilities and invasion histories of the two species. The global colonization of *Ae. aegypti* is older than in *Ae. albopictus* dating to 100 of years ago (Powell et al., 2018). In *Ae. albopictus*, reports of very high levels of differentiation among samples of recently invading populations at regional levels have been identified in Southern Russia, but heavily restricted gene flow or population exchange is reported between the different study sites (Konorov et al., 2021). The weak genetic structure, high levels of admixture, and lack of IBD found in this study for A*e. albopictus* suggest rapid expansion most likely through human‐aided dispersal along transportation routes across the Alps. The human transportation network is known to have influenced and shaped the rapid spread of *Ae. albopictus* at regional levels (for a review see Medley et al., 2015 and Medlock et al., 2015). Switzerland is crossed by the European highways (E35 and E43). The E35 is a south–north European route that runs from Rome (Italy) to Amsterdam (the Netherlands), while the E43 connects Eastern Switzerland with Germany. Our results support the hypothesis that E35 has indeed acted as a key route of introduction of *Ae. albopictus* across the Alps, as previously suggested by surveillance data (Müller et al., 2020).

In Strasbourg, France, we collected a pair of full sibling in two consecutive years at 330 m of distance (Table 4). This finding is an indirect proof of overwintering of a mosquito population north of the Alps as well as the occurrence of skip oviposition behavior. *Aedes albopictus* is adapted to colder temperatures by producing dormant egg stages in fall that overwinter and hatch in the subsequent spring. Diapausing eggs have been described as the main mechanism enabling range expansion into regions at higher latitudes in North America and Northern Europe (for review see Armbruster, 2016; Batz et al., 2020). For overwintering populations, we would expect that mosquitoes caught in spring are closely related to mosquitoes caught in fall from the previous year. In Strasbourg, a full‐sibling pair was collected in two consecutive years supporting the hypothesis that the second pair hatched from eggs laid by the same mother and implying that they must have overwintered as diapausing egg. This result is supported by field observations from the local surveillance program as *Ae. albopictus* individuals were present in the same area in the preceding years, with the first detection dated in 2014 (Krupa et al., 2020), suggesting the presence of a self‐sustaining population. The proportion of the population actually overwintering and the proportion of individuals which are re‐introduced every year remain yet to be identified in this area. In Switzerland, we did not find such closely related siblings, but we observed a high genetic similarity in mosquitoes collected from the same sites in two consecutive years (Figure 7b–d). This, together with the decline of heterozygosity and the increase in the inbreeding coefficient between mosquitoes from the same sites, supports the presence of overwintering populations (Figure 7). A population that is continuously inbreeding locally is likely to have higher inbreeding coefficient as mating occurs between individuals related by descent and an overall decline in heterozygosity is expected (Rumball et al., 1994). The reporting by local surveillance activities of individuals early in the season in both 2018 and 2019 further supports the likely presence of overwintering eggs. In Germany, studies based on ecological (Kuhlisch et al., 2018; Pluskota et al., 2016) and genetic data (Lühken et al., 2020; Walther et al., 2017) also suggest the presence of overwintering populations across the country.

The recovery of one pair of full siblings between two consecutive years in Strasbourg also provides indirect evidence of skip oviposition (Table 4). Skip oviposition describes the behavior of a female mosquito depositing eggs in multiple breeding sites during a single gonotrophic cycle (Corbet & Chadee, 1993). Since the full siblings must have been from the same mother, we can conclude that the same female mosquito laid eggs of a single batch in two different breeding sites. This result confirms previous laboratory (Davis et al., 2015) and field (Davis et al., 2016) evidence, showing skip oviposition behavior in this species. This is especially relevant from a control perspective as this behavior could be potentially exploited to develop auto dissemination control measures (Caputo et al., 2012; Gaugler et al., 2012).

The high genetic variability of the mosquito populations (Figure 3, Table 2) across the Alps suggests multiple re‐introductions from different sources. The frequent re‐introductions of specimens from multiple sources are the likely cause of the high level of admixture found in our data (Figure 3), which is also contributing to maintain high genetic variation within local populations. This, in turn, might increase the probability of further spread. We found evidence of eggs going through diapause across the Alps, which suggests that the mosquito is potentially adapted to survive the colder winters. Taken together, the expansion patterns suggest that the Alps are not a barrier for *Ae. albopictus* and we may expect further spread in Central Europe. As a consequence, control measures should be designed to detect and target mosquitoes early in the season in order to prevent adults from hatching from diapausing eggs.

## AUTHOR CONTRIBUTIONS

**Laura Vavassori:** Conceptualization (equal); data curation (lead); formal analysis (lead); investigation (lead); methodology (lead); project administration (lead); resources (lead); writing – original draft (lead); writing – review and editing (lead). **Ann‐Christin Honnen:** Data curation (equal); formal analysis (equal); methodology (equal); supervision (equal); validation (supporting); visualization (supporting); writing – review and editing (equal). **Norah Saarman:** Data curation (equal); formal analysis (equal); methodology (equal); resources (equal); software (lead); writing – review and editing (equal). **Adalgisa Caccone:** Formal analysis (equal); investigation (equal); methodology (equal); resources (equal); software (equal); supervision (equal); writing – review and editing (equal). **Pie Müller:** Conceptualization (lead); funding acquisition (lead); investigation (equal); methodology (equal); supervision (lead); visualization (equal); writing – review and editing (equal).

## CONFLICT OF INTEREST

The authors declare no competing interests.

## Supporting information


Appendix S1

## Data Availability

Raw data for this study are available on Dryad Digital Repository https://doi.org/10.5061/dryad.jm63xsjdr.

## References

[ece39138-bib-0001] Adhami, J. , & Reiter, P. (1998). Introduction and establishment of *Aedes* (Stegomyia) *albopictus* skuse (Diptera: Culicidae) in Albania. American Mosquito Control Association, 14(3), 340–343. 9813831

[ece39138-bib-0002] Alexander, D. H. , Novembre, J. , & Lange, K. (2009). Fast model‐based estimation of ancestry in unrelated individuals. Genome Research, 19(9), 1655–1664. 10.1101/gr.094052.109 19648217 PMC2752134

[ece39138-bib-0003] Armbruster, P. A. (2016). Photoperiodic diapause and the establishment of *Aedes albopictus* (Diptera: Culicidae) in North America. Journal of Medical Entomology, 53(5), 1013–1023. 10.1093/jme/tjw037 27354438 PMC5013814

[ece39138-bib-0004] Battaglia, V. , Gabrieli, P. , Brandini, S. , Capodiferro, M. R. , Javier, P. A. , Chen, X. G. , Achilli, A. , Semino, O. , Gomulski, L. M. , Malacrida, A. R. , Gasperi, G. , Torroni, A. , & Olivieri, A. (2016). The worldwide spread of the tiger mosquito as revealed by mitogenome haplogroup diversity. Frontiers in Genetics, 7, 208. 10.3389/fgene.2016.00208 27933090 PMC5120106

[ece39138-bib-0005] Batz, Z. A. , Clemento, A. J. , Fritzenwanker, J. , Ring, T. J. , Garza, J. C. , & Armbruster, P. A. (2020). Rapid adaptive evolution of the diapause program during range expansion of an invasive mosquito. Evolution, 74(7), 1451–1465. 10.1111/evo.14029 32490563 PMC8023039

[ece39138-bib-0006] Becker, N. , Geier, M. , Balczun, C. , Bradersen, U. , Huber, K. , Kiel, E. , Kruger, A. , Luhken, R. , Orendt, C. , Plenge‐Bonig, A. , Rose, A. , Schaub, G. A. , & Tannich, E. (2013). Repeated introduction of *Aedes albopictus* into Germany, July to October 2012. Parasitology Research, 112(4), 1787–1790. 10.1007/s00436-012-3230-1 23242268

[ece39138-bib-0007] Biebinger, S. (2020). Uberwachung und Bekampfung der Asiatischen Tigermucke im Kanton Basel‐Stadt 2019. Kantonales Laboratorium BS.

[ece39138-bib-0008] Birungi, J. , & Munstermann, L. E. (2002). Genetic structure of *Aedes albopictus* (Diptera: Culicidae) populations based on mitochondrial ND5 sequences: Evidence for an independent invasion into Brazil and United States. Annals of the Entomological Society of America, 95(1), 125–132. 10.1603/0013-8746

[ece39138-bib-0009] Bonizzoni, M. , Gasperi, G. , Chen, X. G. , & James, A. A. (2013). The invasive mosquito species *Aedes albopictus*: current knowledge and future perspectives. Trends in Parasitology, 29(9), 460–468. 10.1016/j.pt.2013.07.003 23916878 PMC3777778

[ece39138-bib-0010] Bowcock, A. M. , Ruiz‐Linares, A. , Tomfohrde, J. , Minch, E. , Kidd, J. R. , & Cavalli‐Sforza, L. L. (1994). High resolution of human evolutionary trees with polymorphic microsatellites. Nature, 368(6470), 455–457. 10.1038/368455a0 7510853

[ece39138-bib-0011] Caminade, C. , Medlock, J. M. , Ducheyne, E. , McIntyre, K. M. , Leach, S. , Baylis, M. , & Morse, A. P. (2012). Suitability of European climate for the Asian tiger mosquito *Aedes albopictus*: recent trends and future scenarios. Journal of the Royal Society Interface, 9(75), 2708–2717. 10.1098/rsif.2012.0138 22535696 PMC3427500

[ece39138-bib-0012] Cancrini, G. , di Regalbono, A. F. , Ricci, I. , Tessarin, C. , Gabrielli, S. , & Pietrobelli, M. (2003). *Aedes albopictus* is a natural vector of Dirofilaria immitis in Italy. Veterinary Parasitology, 118(3–4), 195–202. 10.1016/j.vetpar.2003.10.011 14729167

[ece39138-bib-0013] Caputo, B. , Ienco, A. , Cianci, D. , Pombi, M. , Petrarca, V. , Baseggio, A. , Devine, G. J. , & della Torre, A. (2012). The “auto‐dissemination” approach: A novel concept to fight *Aedes albopictus* in Urban Areas. PLoS Neglected Tropical Diseases, 6(8), e1793. 10.1371/journal.pntd.0001793 22953015 PMC3429402

[ece39138-bib-0014] Catchen, J. , Bassham, S. , Wilson, T. , Currey, M. , O'Brien, C. , Yeates, Q. , & Cresko, W. A. (2013). The population structure and recent colonization history of Oregon threespine stickleback determined using restriction‐site associated DNA‐sequencing. Molecular Ecology, 22(11), 2864–2883. 10.1111/mec.12330 23718143 PMC3712868

[ece39138-bib-0015] Chang, C. C. , Chow, C. C. , Tellier, L. , Vattikuti, S. , Purcell, S. M. , & Lee, J. J. (2015). Second‐generation PLINK: rising to the challenge of larger and richer datasets. GigaScience, 4, 7. 10.1186/s13742-015-0047-8 25722852 PMC4342193

[ece39138-bib-0016] Chen, K. Y. , Marschall, E. A. , Sovic, M. G. , Fries, A. C. , Gibbs, H. L. , & Ludsin, S. A. (2018). AssignPop: An R package for population assignment using genetic, non‐genetic, or integrated data in a machine‐learning frame‐work. Methods in Ecology and Evolution, 9(2), 439–446.

[ece39138-bib-0017] Corbet, P. S. , & Chadee, D. D. (1993). An improved method for detecting substrate preferences shown by mosquitoes that exhibit ‘skip oviposition’. Physiological Entomology, 18(2), 114–118. 10.1111/j.1365-3032.1993.tb00457.x

[ece39138-bib-0018] Cristescu, M. E. (2015). Genetic reconstructions of invasion history. Molecular Ecology, 24(9), 2212–2225. 10.1111/mec.13117 25703061

[ece39138-bib-0019] Dalla Pozza, G. , & Majori, G. (1992). First record of *Aedes albopictus* establishment in Italy. Journal American Mosquito Control Association, 8(3), 318–320. 1402871

[ece39138-bib-0020] Danecek, P. , Auton, A. , Abecasis, G. , Albers, C. A. , Banks, E. , DePristo, M. A. , Handsaker, R. E. , Lunter, G. , Marth, G. T. , Sherry, S. T. , McVean, G. , Durbin, R. , & Genomes Project Analysis Group . (2011). The variant call format and VCFtools. Bioinformatics, 27(15), 2156–2158. 10.1093/bioinformatics/btr330 21653522 PMC3137218

[ece39138-bib-0021] Davis, T. J. , Kaufman, P. E. , Hogsette, J. A. , & Kline, D. L. (2015). The effects of larval habitat quality on *Aedes albopictus* skip oviposition. Journal of the American Mosquito Control Association, 31(4), 321–328. 26675453 10.2987/moco-31-04-321-328.1

[ece39138-bib-0022] Davis, T. J. , Kaufman, P. E. , Tatem, A. J. , Hogsette, J. A. , & Kline, D. L. (2016). Development and evaluation of an attractive self‐marking ovitrap to measure dispersal and determine skip oviposition in *Aedes albopictus* (Diptera: Culicidae) field populations. Journal of Medical Entomology, 53, 31–38. 10.1093/jme/tjv170 26534725 PMC4723682

[ece39138-bib-0023] Dlugosch, K. M. , & Parker, I. M. (2008). Founding events in species invasions: Genetic variation, adaptive evolution, and the role of multiple introductions. Molecular Ecology, 17(1), 431–449. 10.1111/j.1365-294X.2007.03538.x 17908213

[ece39138-bib-0024] ECDC . (2019). *Aedes albopictus*—current known distribution: August 2019. https://www.ecdc.europa.eu/en/publications‐data/aedes‐albopictus‐current‐known‐distribution‐august‐2019

[ece39138-bib-0025] Egizi, A. , Kiser, J. , Abadam, C. , & Fonseca, D. M. (2016). The hitchhiker's guide to becoming invasive: exotic mosquitoes spread across a US state by human transport not autonomous flight. Molecular Ecology, 25(13), 3033–3047. 10.1111/mec.13653 27087539

[ece39138-bib-0026] Estoup, A. , & Guillemaud, T. (2010). Reconstructing routes of invasion using genetic data: why, how and so what? Molecular Ecology, 19(19), 4113–4130. 10.1111/j.1365-294X.2010.04773.x 20723048

[ece39138-bib-0027] Facon, B. , Genton, B. J. , Shykoff, J. , Jarne, P. , Estoup, A. , & David, P. (2006). A general eco‐evolutionary framework for understanding bioinvasions. Trends in Ecology & Evolution, 21(3), 130–135. 10.1016/j.tree.2005.10.012 16701488

[ece39138-bib-0028] Flacio, E. , Engeler, L. , Tonolla, M. , & Müller, P. (2016). Spread and establishment of *Aedes albopictus* in southern Switzerland between 2003 and 2014: an analysis of oviposition data and weather conditions. Parasites & Vectors, 9(1), 304. 10.1186/s13071-016-1577-3 27229686 PMC4882898

[ece39138-bib-0029] Fuehrer, H. P. , Schoener, E. , Weiler, S. , Barogh, B. S. , Zittra, C. , & Walder, G. (2020). Monitoring of alien mosquitoes in Western Austria (Tyrol, Austria, 2018). PLoS Neglected Tropical Diseases, 14(6), e0008433. 10.1371/journal.pntd.0008433 32574163 PMC7337398

[ece39138-bib-0030] Garnas, J. R. , Auger‐Rozenberg, M. A. , Roques, A. , Bertelsmeier, C. , Wingfield, M. J. , Saccaggi, D. L. , Roy, H. E. , & Slippers, B. (2016). Complex patterns of global spread in invasive insects: eco‐evolutionary and management consequences. Biological Invasions, 18(4), 935–952. 10.1007/s10530-016-1082-9

[ece39138-bib-0031] Gaugler, R. , Suman, D. , & Wang, Y. (2012). An autodissemination station for the transfer of an insect growth regulator to mosquito oviposition sites. Medical and Veterinary Entomology, 26(1), 37–45. 10.1111/j.1365-2915.2011.00970.x 21689125

[ece39138-bib-0032] Global Invasive Species Database . (2020). Aedes albopictus . http://www.iucngisd.org/gisd/search.php

[ece39138-bib-0033] Goubert, C. , Minard, G. , Vieira, C. , & Boulesteix, M. (2016). Population genetics of the Asian tiger mosquito *Aedes albopictus*, an invasive vector of human diseases. Heredity, 117(3), 125–134. 10.1038/hdy.2016.35 27273325 PMC4981682

[ece39138-bib-0034] Goudet, J. (2005). Hierfstat, a package for R to compute and test variance components and F‐statistics. Molecular Ecology Notes, 5, 184–186.

[ece39138-bib-0035] Gratz, N. G. (2004). Critical review of the vector status of *Aedes albopictus* . Medical and Veterinary Entomology, 18, 215–227. 10.1111/j.0269-283X.2004.00513.x 15347388

[ece39138-bib-0036] Hanson, S. M. , & Craig, G. B. J. (1994). Cold acclimation, diapause, and geographic origin affect cold hardiness in eggs of *Aedes albopictus* (Diptera: Culicidae). Journal of Medical Entomology, 31(2), 192–201. 8189409 10.1093/jmedent/31.2.192

[ece39138-bib-0037] Hardy, O. J. , & Vekemans, X. (2002). spagedi: a versatile computer program to analyse spatial genetic structure at the individual or population levels. Molecular Ecology Notes, 2(4), 618–620. 10.1046/j.1471-8286.2002.00305.x

[ece39138-bib-0038] Huson, D. H. , & Bryant, D. (2006). Application of phylogenetic networks in evolutionary studies. Molecular Biology and Evolution, 23(2), 254–267. 10.1093/molbev/msj030 16221896

[ece39138-bib-0039] Iacchei, M. , Ben‐Horin, T. , Selkoe, K. A. , Bird, C. E. , García‐Rodríguez, F. J. , & Toonen, R. J. (2013). Combined analyses of kinship and FST suggest potential drivers of chaotic genetic patchiness in high gene‐flow populations. Molecular Ecology, 22(13), 3476–3494. 10.1111/mec.12341 23802550 PMC3749441

[ece39138-bib-0040] Jasper, M. , Schmidt, T. L. , Ahmad, N. W. , Sinkins, S. P. , & Hoffmann, A. A. (2019). A genomic approach to inferring kinship reveals limited intergenerational dispersal in the yellow fever mosquito. Molecular Ecology Resources, 20(6), 1254–1264. 10.1111/1755-0998.13043 PMC6790672 31125998

[ece39138-bib-0041] Jombart, T. , & Ahmed, I. (2011). adegenet 1.3‐1: new tools for the analysis of genome‐wide SNP data. Bioinformatics, 27(21), 3070–3071. 10.1093/bioinformatics/btr521 21926124 PMC3198581

[ece39138-bib-0042] Jombart, T. , Devillard, S. , & Balloux, F. (2010). Discriminant analysis of principal components: a new method for the analysis of genetically structured populations. BMC Genetics, 11(1), 94. 10.1186/1471-2156-11-94 20950446 PMC2973851

[ece39138-bib-0043] Kalinowski, S. , Wagner, A. , & Taper, M. (2006). ML‐RELATE: a computer program for maximum likelihood estimation of relatedness and relationship. Molecular Ecology Notes, 6(2), 576–579. 10.1111/j.1471-8286

[ece39138-bib-0044] Kobayashi, M. , Nihel, N. , & Kurihara, T. (2002). Analysis of northern distribution of *Aedes albopictus* (Diptera: Culicidae) in Japan by geographical information system. Journal of Medical Entomology, 39(1), 4–11. 10.1603/0022-2585-39.1.4 11931270

[ece39138-bib-0045] Konorov, E. A. , Yurchenko, V. , Patraman, I. , Lukashev, A. , & Oyun, N. (2021). The effects of genetic drift and genomic selection on differentiation and local adaptation of the introduced populations of *Aedes albopictus* in southern Russia. PeerJ, 9, e11776. 10.7717/peerj.11776 34327056 PMC8308624

[ece39138-bib-0046] Kotsakiozi, P. , Richardson, J. B. , Pichler, V. , Favia, G. , Martins, A. J. , Urbanelli, S. , Armbruster, P. A. , & Caccone, A. (2017). Population genomics of the Asian tiger mosquito, *Aedes albopictus*: insights into the recent worldwide invasion. Ecology and Evolution, 7(23), 10143–10157. 10.1002/ece3.3514 29238544 PMC5723592

[ece39138-bib-0047] Kraemer, M. U. G. , Reiner, R. C. , Brady, O. J. , Messina, J. P. , Gilbert, M. , Pigott, D. M. , Yi, D. , Johnson, K. , Earl, L. , Marczak, L. B. , Shirude, S. , Weaver, N. D. , Bisanzio, D. , Perkins, T. A. , Lai, S. , Lu, X. , Jones, P. , Coelho, G. E. , Carvalho, R. G. , … Golding, N. (2019). Past and future spread of the arbovirus vectors Aedes aegypti and *Aedes albopictus* . Nature Microbiology, 1, 854–863. 10.1038/s41564-019-0376-y PMC6522366 30833735

[ece39138-bib-0048] Krupa, E. , Schaffner, F. , Bender, C. , & Mathieu, B. (2020). Progression du moustique tigre en France: surveillance transfrontalière dans le Grand‐Est. Revue Francophone des Laboratoires, 2020(524), 53–61. 10.1016/S1773-035X(20)30231-8

[ece39138-bib-0049] Kuhlisch, C. , Kampen, H. , & Walther, D. (2018). The Asian tiger mosquito *Aedes albopictus* (Diptera: Culicidae) in Central Germany: Surveillance in its northernmost distribution area. Acta Tropica, 188, 78–85. 10.1016/j.actatropica.2018.08.019 30145257

[ece39138-bib-0050] Li, H. , & Durbin, R. (2009). Fast and accurate short read alignment with Burrows‐Wheeler transform. Bioinformatics, 25(14), 1754–1760. 10.1093/bioinformatics/btp324 19451168 PMC2705234

[ece39138-bib-0051] Lockwood, J. L. , Cassey, P. , & Blackburn, T. (2005). The role of propagule pressure in explaining species invasions. Trends in Ecology & Evolution, 20(5), 223–228. 10.1016/j.tree.2005.02.004 16701373

[ece39138-bib-0052] Loiselle, B. A. , Sork, V. L. , Nason, J. , & Graham, C. (1995). Spatial genetic structure of a tropical understory shrub, Psychotria officinalis (Rubiaceae). American Journal of Botany, 82(11), 1420–1425. 10.1002/j.1537-2197.1995.tb12679.x

[ece39138-bib-0053] Lombaert, E. , Guillemaud, T. , Cornuet, J. M. , Malausa, T. , Facon, B. , & Estoup, A. (2010). Bridgehead effect in the worldwide invasion of the biocontrol harlequin ladybird. PLoS One, 5(3), e9743. 10.1371/journal.pone.0009743 20305822 PMC2840033

[ece39138-bib-0054] Lühken, R. , Heitmann, A. , Jansen, S. , Schmidt‐Chanasit, J. , Borstler, J. , Werner, D. , Kampen, H. , Kuhn, C. , Pluskota, B. , Ferstl, I. , Jost, A. , Becker, N. , & Tannich, E. (2020). Microsatellite typing of *Aedes albopictus* (Diptera: Culicidae) populations from Germany suggests regular introductions. Infection, Genetics and Evolution, 81, 104237. 10.1016/j.meegid.2020.104237 32045712

[ece39138-bib-0055] Malinsky, M. , Trucchi, E. , Lawson, D. J. , & Falush, D. (2018). RADpainter and fineRADstructure: Population Inference from RADseq Data. Molecular Biology and Evolution, 35(5), 1284–1290. 10.1093/molbev/msy023 29474601 PMC5913677

[ece39138-bib-0056] Manichaikul, A. , Mychaleckyj, J. C. , Rich, S. S. , Daly, K. , Sale, M. , & Chen, W. M. (2010). Robust relationship inference in genome‐wide association studies. Bioinformatics, 26(22), 2867–2873. 10.1093/bioinformatics/btq559 20926424 PMC3025716

[ece39138-bib-0057] Manni, M. , Guglielmino, C. R. , Scolari, F. , Vega‐Rua, A. , Failloux, A. B. , Somboon, P. , Lisa, A. , Savini, G. , Bonizzoni, M. , Gomulski, L. M. , Malacrida, A. R. , & Gasperi, G. (2017). Genetic evidence for a worldwide chaotic dispersion pattern of the arbovirus vector, *Aedes albopictus* . PLoS Neglected Tropical Diseases, 11(1), e0005332. 28135274 10.1371/journal.pntd.0005332 PMC5300280

[ece39138-bib-0058] Mantel, N. (1967). The detection of disease clustering and a generalized regression approach. Cancer Research, 27(2), 209–220. 6018555

[ece39138-bib-0059] Medley, K. A. , Jenkins, D. G. , & Hoffman, E. A. (2015). Human‐aided and natural dispersal drive gene flow across the range of an invasive mosquito. Molecular Ecology, 24(2), 284–295. 10.1111/mec.12925 25230113

[ece39138-bib-0060] Medlock, J. M. , Hansford, K. M. , Versteirt, V. , Cull, B. , Kampen, H. , Fontenille, D. , Hendrickx, G. , Zeller, H. , Van Bortel, W. , & Schaffner, F. (2015). An entomological review of invasive mosquitoes in Europe. Bulletin of Entomological Research, 105(6), 637–663. 10.1017/S0007485315000103 25804287

[ece39138-bib-0061] Müller, P. , Engeler, L. , Vavassori, L. , Suter, T. , Guidi, V. , Gschwind, M. , Tonolla, M. , & Flacio, E. (2020). Surveillance of invasive Aedes mosquitoes along Swiss traffic axes reveals different dispersal modes for *Aedes albopictus* and *Ae. japonicus* . PLoS Neglected Tropical Diseases, 14(9), e0008705. 10.1371/journal.pntd.0008705 32986704 PMC7544034

[ece39138-bib-0062] Nazareno, A. G. , Bemmels, J. B. , Dick, C. W. , & Lohmann, L. G. (2017). Minimum sample sizes for population genomics: An empirical study from an Amazonian plant species. Molecular Ecology Resources, 17(6), 1136–1147. 10.1111/1755-0998.12654 28078808

[ece39138-bib-0063] O'Leary, S. J. , Puritz, J. B. , Willis, S. C. , Hollenbeck, C. M. , & Portnoy, D. S. (2018). These aren't the loci you'e looking for: Principles of effective SNP filtering for molecular ecologists. Molecular Ecology, 27(16), 3193–3206. 10.1111/mec.14792 29987880

[ece39138-bib-0064] Oswaldo, P. F. (1986). Identificação de Aedes (Stegomyia) albopictus (Skuse) no Brasil. Revista de Saúde Pública, 20, 244–245. 3809982 10.1590/s0034-89101986000300009

[ece39138-bib-0065] Palatini, U. , Masri, R. A. , Cosme, L. V. , Koren, S. , Thibaud‐Nissen, F. , Biedler, J. K. , Krsticevic, F. , Johnston, J. S. , Halbach, R. , Crawford, J. E. , Antoshechkin, I. , Failloux, A. , Pischedda, E. , Marconcini, M. , Ghurye, J. , Rhie, A. , Sharma, A. , Karagodin, D. A. , … Jenrette, J. (2020). Improved reference genome of the arboviral vector *Aedes albopictus* . Genome Biology, 21, 215. 10.1186/s13059-020-02141-w 32847630 PMC7448346

[ece39138-bib-0066] Paupy, C. , Delatte, H. , Bagny, L. , Corbel, V. , & Fontenille, D. (2009). *Aedes albopictus*, an arbovirus vector: From the darkness to the light. Microbes and Infection, 11, 1177–1185. 10.1016/j.micinf.2009.05.005 19450706

[ece39138-bib-0067] Pichler, V. , Kotsakiozi, P. , Caputo, B. , Serini, P. , Caccone, A. , & Torre, A. D. (2019). Complex interplay of evolutionary forces shaping population genomic structure of invasive *Aedes albopictus* in southern Europe. PLoS Neglected Tropical Diseases, 13(8), e0007554. 10.1371/journal.pntd.0007554 31437154 PMC6705758

[ece39138-bib-0068] Pluskota, B. , Jöst, A. , Augsten, X. , Stelzner, L. , Ferstl, I. , & Becker, N. (2016). Successful overwintering of *Aedes albopictus* in Germany. Parasitology Research, 115(8), 3245–3247. 10.1007/s00436-016-5078-2 27112761

[ece39138-bib-0069] Pluskota, B. , Storch, V. , Braunbeck, T. , Beck, M. , & Becker, N. (2008). First record of *Stegomyia albopicta* (Skuse) (Diptera: Culicidae) in Germany. European Mosquito Bulletin, 26, 1–5.

[ece39138-bib-0070] Powell, J. R. , Gloria‐Soria, A. , & Kotsakiozi, P. (2018). Recent history of *Aedes aegypti*: vector genomics and epidemiology records. Bioscience, 68(11), 854–860. 10.1093/biosci/biy119 30464351 PMC6238964

[ece39138-bib-0071] R Core Team . (2020). R: A language and environment for statistical computing. R Foundation for Statistical Computing.

[ece39138-bib-0072] Rašić, G. , Filipović, I. , Weeks, A. R. , & Hoffmann, A. A. (2014). Genome‐wide SNPs lead to strong signals of geographic structure and relatedness patterns in the major arbovirus vector, *Aedes aegypti* . BMC Genomics, 15(1), 275. 10.1186/1471-2164-15-275 24726019 PMC4023594

[ece39138-bib-0073] Rius, M. , & Darling, J. A. (2014). How important is intraspecific genetic admixture to the success of colonising populations? Trends in Ecology & Evolution, 29(4), 233–242. 10.1016/j.tree.2014.02.003 24636862

[ece39138-bib-0074] Rumball, W. , Franklin, I. R. , Frankham, R. , & Sheldon, B. L. (1994). Decline in heterozygosity under full‐sib and double first‐cousin inbreeding in drosophila‐melanogaster. Genetics, 136(3), 1039–1049. 8005413 10.1093/genetics/136.3.1039 PMC1205861

[ece39138-bib-0075] Sabatini, A. , Trovato, G. , & Coluzzi, M. (1990). *Aedes albopictus* in Italy and possible diffusion of the species into the Mediterranean area. Parassitologia, 32, 301–304. 2132441

[ece39138-bib-0076] Schmidt, T. L. , Chung, J. , Honnen, A. C. , Weeks, A. R. , & Hoffmann, A. A. (2020). Population genomics of two invasive mosquitoes (*Aedes aegypti* and *Aedes albopictus*) from the Indo‐Pacific. PLoS Neglected Tropical Diseases, 14(7), e0008463. 10.1371/journal.pntd.0008463 32678817 PMC7390453

[ece39138-bib-0077] Schmidt, T. L. , Chung, J. , Van Rooyen, A. , Sly, A. , Weeks, A. R. , & Hoffmann, A. A. (2020). Incursion pathways of the Asian tiger mosquito (*Aedes albopictus*) into Australia contrast sharply with those of the yellow fever mosquito (*Aedes aegypti*). Pest Management Science, 76, 4202–4209. 32592440 10.1002/ps.5977

[ece39138-bib-0078] Schmidt, T. L. , Filipovic, I. , Hoffmann, A. A. , & Rasic, G. (2018). Fine‐scale landscape genomics helps explain the slow spatial spread of Wolbachia through the Aedes aegypti population in Cairns, Australia. Heredity, 120(5), 386–395. 10.1038/s41437-017-0039-9 29358725 PMC5889405

[ece39138-bib-0079] Schmidt, T. L. , Rašić, G. , Zhang, D. , Zheng, X. , Xi, Z. , & Hoffmann, A. A. (2017). Genome‐wide SNPs reveal the drivers of gene flow in an urban population of the Asian Tiger Mosquito, *Aedes albopictus* . PLOS Neglected Tropical Diseases, 11(10), e0006009. 10.1371/journal.pntd.0006009 29045401 PMC5662242

[ece39138-bib-0080] Sherpa, S. , Blum, M. G. B. , Capblancq, T. , Cumer, T. , Rioux, D. , & Després, L. (2019). Unravelling the invasion history of the Asian tiger mosquito in Europe. Molecular Ecology, 28(9), 2360–2377. 10.1111/mec.15071 30849200

[ece39138-bib-0081] Shirk, A. J. , Landguth, E. L. , & Cushman, S. A. (2017). A comparison of individual‐based genetic distance metrics for landscape genetics. Molecular Ecology Resources, 17(6), 1308–1317. 10.1111/1755-0998.12684 28449317

[ece39138-bib-0082] Slatkin, M. (1985). Gene flow in natural populations. Annual Review of Ecology and Systematics, 16(1), 393–430.

[ece39138-bib-0083] Sprenger, D. , & Wuithiranyagool, T. (1986). The discovery and distribution of *Aedes albopictus* in Harris County, Texas. Journal American Mosquito Control Association, 2(2), 217–219. 3507493

[ece39138-bib-0084] Vavassori, L. , Saddler, A. , & Muller, P. (2019). Active dispersal of *Aedes albopictus*: A mark‐release‐recapture study using self‐marking units. Parasites & Vectors, 12(1), 583. 10.1186/s13071-019-3837-5 31831040 PMC6909613

[ece39138-bib-0085] Walther, D. , Scheuch, D. E. , & Kampen, H. (2017). The invasive Asian tiger mosquito *Aedes albopictus* (Diptera: Culicidae) in Germany: Local reproduction and overwintering. Acta Tropica, 166, 186–192. 10.1016/j.actatropica.2016.11.024 27876647

[ece39138-bib-0086] Weir, B. S. , & Cockerham, C. C. (1984). Estimating F‐statistics for the analysis of population structure. Evolution, 38(6), 1358–1370. 10.1111/j.1558-5646.1984.tb05657.x 28563791

[ece39138-bib-0087] Werner, D. , Kronefeld, M. , Schaffner, F. , & Kampen, H. (2012). Two invasive mosquito species, *Aedes albopictus* and *Aedes japonicus* japonicus, trapped in south‐west Germany, July to August 2011. Euro Surveillance, 17(4), 14–17. 10.2807/ese.17.04.20067-en 22297138

[ece39138-bib-0088] Willing, E. M. , Dreyer, C. , & van Oosterhout, C. (2012). Estimates of genetic differentiation measured by F‐ST do not necessarily require large sample sizes when using many SNP markers. PLoS One, 7(8), e42649. 10.1371/journal.pone.0042649 22905157 PMC3419229

[ece39138-bib-0089] Wong, P. S. , Li, M. Z. , Chong, C. S. , Ng, L. C. , & Tan, C. H. (2013). *Aedes* (Stegomyia) *albopictus* (Skuse): A potential vector of Zika virus in Singapore. PLoS Neglected Tropical Diseases, 7(8), e2348. 10.1371/journal.pntd.0002348 23936579 PMC3731215

[ece39138-bib-0090] Zhong, D. B. , Lo, E. , Hu, R. J. , Metzger, M. E. , Cummings, R. , Bonizzoni, M. , Fujioka, K. K. , Sorvillo, T. E. , Kluh, S. , Healy, S. P. , Fredregill, C. , Kramer, V. L. , Chen, X. G. , & Yan, G. Y. (2013). Genetic analysis of invasive *Aedes albopictus* populations in Los Angeles county, California and its potential public health impact. PLoS One, 8(7), e68586. 10.1371/journal.pone.0068586 23861921 PMC3702605

